# Terminal spin labeling of xylotriose strongly affects interactions in the active site of xylanase BcX

**DOI:** 10.1007/s10858-025-00459-w

**Published:** 2025-03-12

**Authors:** Mahin Saberi, René Dekkers, Leonardo Passerini, Martina Huber, Mark Overhand, Marcellus Ubbink

**Affiliations:** 1 Department of Macromolecular Biochemistry, Leiden Institute of Chemistry, Leiden University, Einsteinweg 55, 2333 CC Leiden, The Netherlands; 2 Department of Bio-Organic Synthesis, Leiden Institute of Chemistry, Leiden University, Einsteinweg 55, 2333 CC Leiden, The Netherlands; 3 Department of Physics, Huygens-Kamerlingh Onnes Laboratory, Leiden University, Niels Bohrweg 2, 2333 CA Leiden, The Netherlands

**Keywords:** Xylanase, Spin label, Transient interactions, NMR spectroscopy, Paramagnetic relaxation enhancement, TEMPO

## Abstract

Paramagnetic probes provide long-range distance information and report on minor conformations of biomacromolecules. However, it is important to realize that any probe can affect the system of interest. Here, we report on the effects of attaching a small nitroxide spin label [TEMPO, (2,2,6,6-tetramethylpiperidin-1-yl)oxyl] to xylotriose, a substrate of the enzyme xylanase from *Bacillus circulans* (BcX). BcX has a long and narrow active site cleft accommodating six xylose units and a secondary binding site on its surface. The aim of the study was to probe the interactions of the substrate with the enzyme using paramagnetic relaxation enhancements (PREs). Binding of the substrate to the surface exposed secondary binding site resulted in strong and localized PREs, indicative of well-defined binding. The xylotriose with diamagnetic control tag was still able to bind the active site cleft, though the rate of exchange was reduced relative to that of untagged xylotriose. The substrate with the paramagnetic TEMPO was not able to bind inside the active site cleft. Also, additional interactions on another surface location showed differences between the paramagnetic substrate and the diamagnetic control, despite the minimal chemical differences between TEMPO modified xylotriose and its reduced, diamagnetic counterpart. Our findings underscore the sensitivity of BcX substrate binding to minor substrate modifications. This study serves as a reminder that any probe, including the attachment of a small paramagnetic group, can affect the behavior of the system under investigation. Even the chemical difference between a paramagnetic tag and its diamagnetic control can result in differences in the molecular interactions.

**Supplementary Information:**

The online version contains supplementary material available at 10.1007/s10858-025-00459-w.

## Introduction

Nuclear magnetic resonance (NMR) spectroscopy offers a versatile approach for characterizing protein–ligand interactions and is sensitive to a wide range of ligand affinities, from nanomolar to millimolar concentrations. Several approaches are used to obtain detailed structural, thermodynamic, and kinetic information about protein–ligand systems (Becker et al. 2018). The primary method is the measurement of chemical shift perturbations (CSPs). CSP analysis identifies protein nuclei directly involved in ligand binding and allows for the calculation of equilibrium dissociation constants (Skeens et al. 2023; Williamson 2013). The method identifies nuclei that undergo changes in their magnetic environment due to the presence of the ligand, suggesting proximity but not providing structural data. Paramagnetic relaxation enhancement (PRE) can complement CSP analysis. The method involves measuring the increase in longitudinal or transverse relaxation rates of nuclear spins induced by a paramagnetic species. PREs can provide structural restraints and are also used to detect fleeting interactions in which the nucleus gets close to the paramagnetic species. Due to the strong, inverse sixth-power distance-dependence, the PRE is very large at short distance (Clore et al. 2009; Clore et al. 2007; Liu et al. 2016; Tang et al. 2008). Given the lack of paramagnetic species in most biological molecules, various methods are employed to attach a paramagnetic center, such as a nitroxide radical, transition metal or lanthanide ion, to either the protein or ligand molecule of interest (Canales et al. 2014; John et al. 2006; Köhling et al. 2016; Lee et al. 2020; Miao et al. 2022; Zhuang et al. 2008). Commonly used paramagnetic probes for PRE measurements include stable nitroxide radicals, such as TEMPO [(2,2,6,6-tetramethylpiperidin-1-yl)oxyl] and metal chelators based on EDTA like molecules and cyclens, or metal-binding peptides (Miao et al. 2022; Nitsche et al. 2018).

**Fig. 1:**
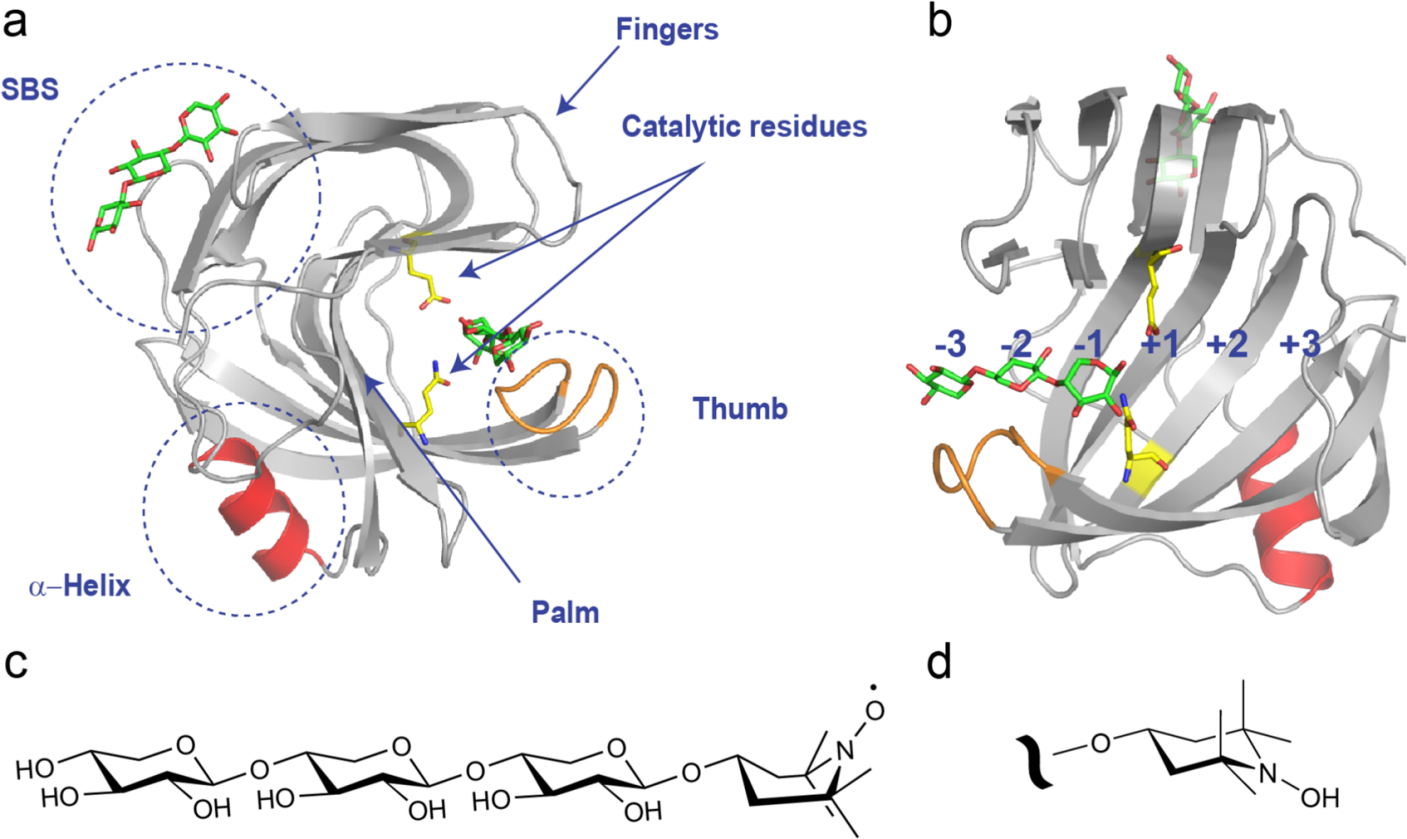
Overall structure of BcX. **a**, **b** Crystal structure of BcX E78Q in complex with xylotriose [PDB code: 8QXY, (Saberi et al. 2024)], depicting the β-jelly roll fold, characterized by thumb, palm and finger domains, as well as the SBS and α-helix. Xylotrioses and the two catalytic residues at positions 78 and 172 are in sticks. A detailed view of the substrate-binding cleft in BcX, highlighting xylotriose bound to the -1, -2, and -3 subsites is shown in (**b**); **c** Chemical structure of the spin-labeled xylotriose (TEMPO-X3); **d** Reduction of the paramagnetic nitroxide (N–O) moiety produces the diamagnetic hydroxylamine (N–OH) derivative. The boat and chair structures of TEMPO are based on experimental observations (see text)

*Bacillus circulans* xylanase (BcX), a member of glycoside hydrolase family 11, functions as a retaining endo-1,4-β-xylanase, cleaving xylan, a polymer of xylose units. It features a β-jelly-roll structure that resembles a right-hand fist (Wakarchuk et al. 1994). The central ‘palm’ region, a twisted beta sheet, forms a narrow groove in which the multimeric substrate binds. A loop linking two beta strands mimics a thumb, and additional β-sheets folded under the palm region mimic ‘fingers’ (Fig. 1a). This structural arrangement creates a hydrophobic core that enhances the stability of the palm region (Paës et al. 2012; Törrönen et al. 1994, 1997). The active site cleft encompasses three negative ( −) and three positive ( +) subsites, with at the center a catalytic dyad, composed of two glutamic acid residues (E78 and E172) (Fig. 1b) (Davies et al. 1997; Törrönen et al., 1997). The enzymatic cleavage of glycosidic bonds demands that the substrate at least spans the − 2, − 1, and + 1 subsites (Paës et al. 2012). The presence of a secondary binding site (SBS) on the surface of the enzyme has been shown through NMR spectroscopy and x-ray crystallography for the GH11 xylanases from *B. circulans*, *B. subtilis*, and *Aspergillus niger* (Ludwiczek et al. 2007; Vandermarliere et al. 2008). Modification of the SBS resulted in up to a three-fold decrease in the activity of the enzymes for xylan, highlighting the importance of SBS for the efficiency of hydrolysis (Cuyvers et al. 2011).

The crystallographic studies provide static images that suggest preferred, ordered binding of the substrate in the − 3, − 2, and − 1 subsites. However, ligand binding in the solution state is more complex and dynamic. In our previous study, NMR titrations with xylose substrates of different lengths revealed nonlinear chemical shift trajectories for resonances of active site nuclei, indicative of at least two binding sites (Saberi et al. 2024). Active site binding can be modeled with a 2:1 model with dissociation constants in the low and high millimolar range. This led us to question whether substrate binding might occur in different orientations or if sliding movements along the active site cleft are possible. To characterize transient interactions of the substrate within the active site, we prepared derivatives of xylotriose (X3) with a TEMPO spin-label covalently attached to the reducing end of the sugar (Para-X3), as well as the nonparamagnetic xylotriose analogue (Dia-X3), obtained by reducing the nitroxide radical with sodium ascorbate (Fig. 1c, d). To our surprise, the TEMPO has major effects on the interactions, and even significant differences between the diamagnetic and paramagnetic forms are observed. It is concluded that the influence of the paramagnetic probe is such that it is difficult to learn more about substrate-enzyme interactions. However, we believe it is of interest to report these results for the bioNMR community, as it demonstrates how significant the disturbance of a small probe can be in specific cases.

## Material and methods

### Chemical synthesis and instrumentation (NMR and EPR)

All reagents were used as purchased unless stated otherwise. Xylotriose and TEMPOL were bought from Megazyme and the Cayman Chemical Company, respectively. Solvents were dried *in vacuo* and stored over 3 Å molecular sieves. Reaction progress was monitored using thin layer chromatography (TLC) on aluminum sheets coated with silica gel 60 F_254_ (Merck). Detection of compounds was done using UV-absorption or by spraying with a solution of (NH_4_)_4_Ce(SO_4_)_4_⋅H_2_O (10 g/mL) and (NH_4_)_6_Mo_7_O_24_⋅H_2_O (25 g/L) in 10% sulfuric acid, followed by heating. For column chromatography, silica gel 60 M (0.04–0.063 mm) was used in combination with indicated solvents and gradient. ^1^H-NMR and ^13^C-NMR spectra were recorded on a Bruker AV-400 or Bruker AV-850 spectrometer in the given solvent. Chemical shifts are given in ppm (δ) relative to the solvent signal or tetramethylsilane (TMS) as internal standard. Given ^13^C-NMR spectra are all decoupled. Abbreviations used for describing signal patterns are: s (singlet), d (doublet), t (triplet), q (quartet), m (multiplet) or b (broad). High-resolution mass spectra (HR-MS) were recorded using a LTQ orbitrap (Thermo Finnigan) equipped with an electron spray ion source in positive mode (source voltage 3.5 kV, sheath gas flow 10 mL/min, capillary temperature 250 °C) with a resolution of 60,000 at m/z (400 mass range m/z = 150–2000) and dioctyl phthalate (m/z = 391.284) as lock mass. The high-resolution mass spectrometer was calibrated prior to measurements with a calibration mixture (Thermo Finnigan). Liquid chromatography mass spectrometry (LC–MS) results were recorded on a LCQ Advantage Max (Thermo Finnigan) ion-trap spectrometer (ESI +) coupled to a Surveyor HPLC system (Thermo Finnigan) equipped with a C_18_ column (Gemini, 4.5 mm × 50 mm, 3 µM particle size, Phenomenex) using buffers A: H_2_O and B: acetonitrile (ACN) or an Agilent technologies 1260 infinity LC–MS with a 6120 Quadrupole MS system equipped with buffers A: H_2_O, B: acetonitrile (ACN) and C: 100 mM NH_4_OAc. EPR spectra were recorded at room temperature with an EMX Plus EPR spectrometer (Bruker BioSpin, Germany) equipped with a SHQ resonator. EPR measurement conditions were microwave frequency: 9.88 GHz, modulation frequency: 100 kHz, modulation amplitude: 0.3 mT, time constant: 20.48 ms, power: 10.02 mW, measurement time: two min. Glass micropipettes of a volume of 50 µL (Blaubrand Intramark, Wertheim, Germany) were filled with 20 µL of sample for each measurement.

#### ***2,3,4-tri-O-acetyl-β-d-xylopyranosyl-(1 → 4)-2,3-di-O-acetyl-β-d-xylopyranosyl-(1 → 4)-1,2,3-tri-O-acetyl-d-xylopyranoside***


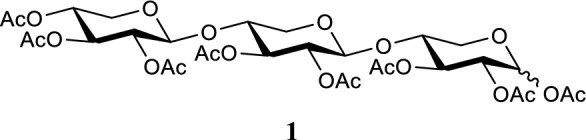


Xylotriose (0.828 g, 2.0 mmol) was cooled to 0 °C. Acetic anhydride (2.46 mL, 26 mmol, 13 eq.) and pyridine (2.09 mL, 26 mmol, 13 eq.) were added. The reaction was stirred for 24 h at 0 °C. Remaining acetic anhydride and pyridine were removed under reduced pressure. The mixture was dissolved in EtOAc and washed three times with 1 M HCl solution, once with aqueous saturated sodium bicarb solution and once with brine. The organic phase was dried using Na_2_SO_4_, filtered and concentrated under reduced pressure. The residue was purified using silica flash column chromatography with a 10:90 → 50:50 EtOAc:*n*-Pent gradient (R_f_ = 0.27 1:1 EtOAc:*n*-Pent). Compound **1** was obtained as a transparent colorless oil (1.50 g, 2.0 mmol, α:β ratio of 4:5 with quantitative yield). ^1^H NMR (400.130 MHz, CDCl_3_, 293 K): δ = 6.22 (d, *J* = 4.0 Hz, 1H), 5.66 (d, *J* = 7.2 Hz, 1H), 5.10 (q, *J* = 7.6, 8.4 Hz, 4H), 4.99–4.94 (m, 2H), 4.88 (td, *J* = 4.4, 3.2 Hz, 2H), 4.78 (m, 3H), 4.58 (d, *J* = 6.0 Hz, 1H), 4.57 (d, *J* = 5.6 Hz, 1H), 4.50 (d, *J* = 6.8 Hz, 1H), 4.49 (d, *J* = 6.8 Hz, 1H), 4.10 (m, 2H), 3.98 (m, 3H), 3.82 (m, 4H), 3.67 (m, 1H), 3.50–3.30 (m, 5H), 2.10 (s, 6H), 2.05 (s, 36H). ^13^C NMR (100.613 MHz, CDCl_3_, 293 K): δ = 170.0, 169.9, 169.8, 169.7, 169.6, 169.4, 169.2, 169.0, 100.9, 100.4, 99.3, 92.3, 89.2, 75.8, 74.9, 74.3, 72.0, 71.0, 70.9, 70.3, 70.2, 70.0, 69.7, 69.4, 68.2, 63.4, 62.6, 61.5, 61.3, 21.5, 20.9, 20.8, 20.6, 20.5. HR-MS (ESI): *m/z* 773.211 [M + Na]^+^, calcd. [C_31_H_42_O_21_ + Na]^+^ 773.211.

#### 2,3,4-tri-O-acetyl-β-d-xylopyranosyl-(1 → 4)-2,3-di-O-acetyl-β-d-xylopyranosyl-(1 → 4)-2,3-di-O-acetyl-d-xylopyranoside


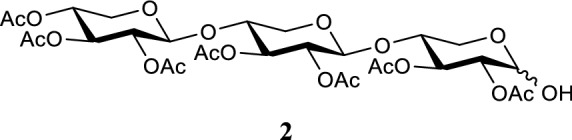


Compound **1** (1.64 g, 2.2 mmol) was dissolved in THF (0.2 M, 11 mL) and 3-(dimethylamino)-1-propylamine (0.55 mL, 4.4 mmol, 2 eq.) was added. The reaction was stirred at room temperature and followed by TLC. When an undesired byproduct was detected, the reaction was diluted with DCM. The mixture was washed with 1 M HCl solution, sodium bicarb and brine. The organic phase was dried Na_2_SO_4_, filtered and concentrated under reduced pressure. Silica flash column chromatography was used for purification with a 40:60 → 70:30 EtOAc:*n*-Pent gradient (R_f_ = 0.31 50:50 EtOAc:*n*-Pent). Compound **2** was obtained as a transparent colorless oil (0.979 g, 1.38 mmol, 63%). ^1^H NMR (400.130 MHz, CDCl_3_, 293 K): δ = 5.44 (t, *J* = 8.4 Hz, 1H), 5.13–5.06 (m, 2H), 4.89 (td, *J* = 4.4, 2.8 Hz, 1H), 4.84–4.74 (m, 3H), 4.58 (d, *J* = 7.2 Hz, 1H), 4.50 (d, *J* = 6.0 Hz, 1H), 4.11 (dd, *J* = 4.8, 7.6 Hz, 1H), 4.02–3.94 (m, 1H), 3.88–3.75 (m, 3H), 3.69 (m, 1H), 3.44–3.31 (m, 2H), 2.07 (s, 21H). ^13^C NMR (100.613 MHz, CDCl_3_, 293 K): δ = 171.0, 170.4, 170.0, 169.9, 169.8, 169.5, 169.2, 100.6, 99.5, 90.4, 74.3, 71.9, 71.3, 71.1, 70.2, 69.9, 68.3, 62.4, 61.6, 20.7. HR-MS (ESI): *m/z* 731.201 [M + Na]^+^, calcd. [C_29_H_40_O_20_ + Na]^+^ 731.201.

#### 2,3,4-tri-O-acetyl-β-d-xylopyranosyl-(1 → 4)-2,3-di-O-acetyl-β-d-xylopyranosyl-(1 → 4)-2,3-di-O-acetyl-N-phenyl-trifluoroacetimidoyl-d-xylopyranoside


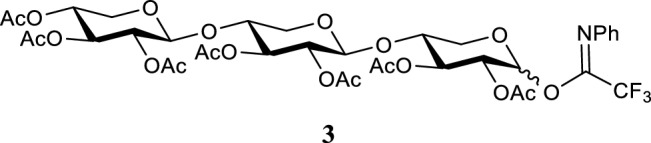


Compound **2** (0.572 g, 0.81 mmol) was co-evaporated three times using distilled toluene and dissolved in dry DCM (0.2 M). While under nitrogen atmosphere, the chloro imidate reagent (0.251 g, 1.21 mmol, 1.5 eq.) and cesium carbonate (0.395 g, 1.21 mmol, 1.5 eq.) were added. The reaction was stirred at room temperature and followed by TLC. After 6 h the reaction was diluted with DCM, filtered over celite and purified by silica flash column chromatography using an EtOAc:*n*-Pent gradient of 10:90 → 50:50 (R_f_ = 0.51 50:50 EtOAc:*n*-Pent). Compound **3** was obtained as a white solid (0.527 g, 0.60 mmol, 74%). ^1^H NMR (400.130 MHz, CDCl_3_, 293 K): δ = 7.34–7.12 (m, 3H), 6.83 (m, 2H), 5.11 (m, 2H), 4.93–4.87 (m, 1H), 4.85–4.76 (m, 2H), 4.59 (t, *J* = 5.6 Hz, 1H), 4.53 (d, *J* = 7.2 Hz, 1H), 4.15–4.10 (m, 2H), 4.01–3.96 (m, 1H), 3.89–3.82 (m, 2H), 3.45–3.32 (m, 2H), 2.11–2.04 (m, 21H). ^13^C NMR (100.613 MHz, CDCl_3_, 293 K): δ = 170.0, 169.9, 169.8, 169.5, 169.4, 169.2, 128.9, 119.4, 100.4, 99.5, 74.3, 73.3, 72.2, 71.0, 70.2, 69.5, 68.2, 62.8, 62.0, 61.5, 20.7. HR-MS (ESI): *m/z* 902.230 [M + Na]^+^, calcd. [C_29_H_40_O_20_ + Na]^+^ 902.230.

#### ***2,3,4-tri-O-acetyl-β-d-xylopyranosyl-(1 → 4)-2,3-di-O-acetyl-β-d-xylopyranosyl-(1 → 4)-2,3-di-O-acetyl-1-TEMPOL-β-d-xylopyranoside***


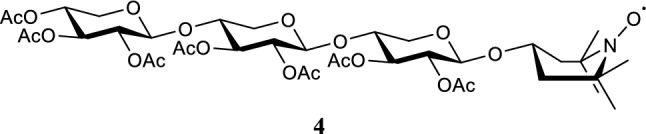


Compound **3** (1.553 g, 1.77 mmol) and TEMPOL (0.456 g, 2.65 mmol, 1.5 eq.) were combined and co-evaporated three times with distilled toluene. While under nitrogen atmosphere, the mixture was dissolved in dry DCM (0.2 M). The mixture was stirred for 30 min, after which it was cooled to –115 °C by using a liquid nitrogen/EtOH cooling bath. TMS-OTf (0.03 mL, 0.18 mmol, 0.1 eq.) was added in a stock solution with dry DCM after which the reaction was gradually warmed up to room temperature, while being followed by TLC. After all starting material had reacted, the reaction was quenched by adding a droplet of triethylamine. The mixture was washed with saturated aqueous sodium bicarb and brine solutions. The organic phase was dried using MgSO_4_, filtered, and concentrated. The resulting crude was purified using silica flash column chromatography with a 20:80 → 60:40 EtOAc:*n*-Pent gradient (R_f_ = 0.21 25:75 EtOAc:*n*-Pent). Compound **4** (1.461 g, 1.69 mmol, 96%) was obtained as a red colored solid. HR-MS (ESI): *m/z* 885.321 [M + Na]^+^, calcd. [C_29_H_40_O_20_ + Na]^+^ 885.324.

#### β-d-xylopyranosyl-(1 → 4)-β-d-xylopyranosyl-(1 → 4)-1-(1-O-4-hydroxy-3,3,5,5-tetramethylpiperidine)-β-d-xylopyranoside


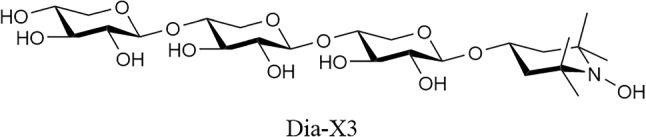


Compound **4** (1.329 g, 1.5 mmol) was dissolved in MeOH (0.2 M), after which 1.99 mL 4.28 M (5 eq.) NaOMe solution was added. The reaction was stirred for 30 min at room temperature, after which quenching was performed by adding AcOH until the pH of the mixture was 7. The mixture was diluted with demi water and extracted thrice with 10 mL DCM. The aqueous phase was concentrated, and the resulting product was purified using HPLC. 0.424 g (≈50%) of material was obtained of which 8.8% was paramagnetic active as determined by EPR measurement. 0.081 g (0.12 mmol) of the obtained product was therefore dissolved in demi water (0.2 M) and treated with ascorbic acid (0.217 g, 1.20 mmol, 10 eq.). Upon full conversion to the diamagnetic compound based on LC–MS and EPR measurements, the mixture was concentrated to dryness after stirring for three hours. The obtained solid was then purified over C_18_ column using a H_2_O:ACN gradient of 100:0 → 95:5 → 90:10 → 50:50. This resulted in 22.8 mg (0.04 mmol, α:β ratio of 1:2 with 33% yield) of Dia-X3 as a white solid. ^1^H NMR (850.130 MHz, D_2_O, 293 K): δ = 4.46 (d, *J* = 7.7 Hz, 1H), 4.40 (d, *J* = 7.7 Hz, 1H), 4.38 (d, *J* = 8.5 Hz, 1H), 4.14–4.08 (m, 1H), 4.02 (dd, *J* = 6.0, 6.8 Hz, 1H), 3.99 (dd, *J* = 5.1, 6.8 Hz, 1H), 3.89 (dd, *J* = 6.0, 6.0 Hz, 1H), 3.72–3.67 (m, 3H), 3.56–3.52 (m, 1H), 3.48–3.45 (m, 2H), 3.35 (t, *J* = 9.4 Hz, 1H), 3.31–3.28 (m, 2H), 3.24–3.15 (m, 5H), 2.10–2.01 (bd, 2H), 1.57–1.46 (bd, 2H), 1.17 (s, 12H). ^13^C NMR (213.765 MHz, D_2_O, 293 K): δ = 102.7, 102.6, 102.4, 77.3, 77.2, 76.5, 74.7, 74.6, 73.8, 73.7, 73.6, 72.1, 66.1, 63.9, 63.8. HR-MS (ESI): *m/z* 570.275 [M + H]^+^, calcd. [C_24_H_43_NO_14_ + H]^+^ 570.275.

#### β-d-xylopyranosyl-(1 → 4)-β-d-xylopyranosyl-(1 → 4)-1-TEMPOL-β-d-xylopyranoside


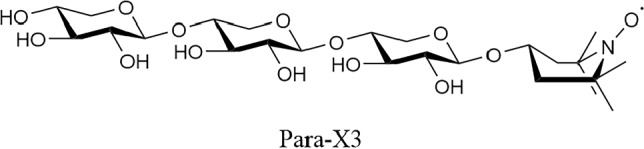


Dia-X3 (6.5 mg, 11.0 µmol) was dissolved in a minimal amount of demi water (22 µL, 0.5 M). MnO_2_ (4.8 mg, 55.2 µmol, 5 eq.) was added and the reaction was stirred for 40 min. After full conversion was observed by LC–MS, the mixture was filtered thrice using PTFE/L 0.45 µm filters. Remaining MnO_2_ was removed using a centrifuge and carefully pipetting the supernatant, which contained Para-X3. After concentrating the sample 4.4 mg (7.70 µmol, 70%) of Para-X3 was obtained as a brown-colored solid. At least 90% of the produced Para-X3 was in the paramagnetic state, based on MS, NMR and EPR data (Figs. S1–S3). HR-MS (ESI): *m/z* 568.260 [M]^+^, calcd. [C_24_H_42_NO_14_]^+^ 568.260.

#### Site-directed mutagenesis and protein production

Site-directed mutagenesis, protein production and purification were performed as described (Saberi et al. 2024).

### Protein NMR spectroscopy

Solutions of ^15^N-labeled BcX E78Q and BcX E78Q-Y69A (100 µM) were titrated with Dia-X3 and Para-X3. Concentrations of up to 50 mM for Dia-X3 and 8 mM for Para-X3 were used. Samples were prepared separately, and each contained 25 mM sodium acetate buffer (pH 5.8) and 10% D_2_O for lock. ^1^H − ^15^N HSQC spectra were obtained on a Bruker HD Avance 850 MHz spectrometer with a cryoprobe, at 20 °C, using 3 mm tubes. In one experiment Para-X3 was reduced in the NMR tube with ascorbic acid to study the effect of reduction in a single sample. The assignment, processing, and analysis of the HSQC spectra were conducted as detailed before (Saberi et al. 2024).

Spectra were processed with Topspin (Bruker) and analyzed using CCPN Analysis v.2.4.2 (Vranken et al. 2005). The weighted average chemical shift perturbations (CSP) for backbone amides (Δδ_avg_) was calculated according to Eq. 1 (modified after Williamson 2013):

1 $$\Delta \delta_{avg} = \sqrt {\delta H^{2} + \frac{\delta N}{{25}}^{2} }$$

where δH and δN are the CSPs for ^1^H and ^15^N nuclei. Titration data were fitted to a 1:1 model Eq. (2), which simplifies to (3) when the ligand concentration [L_t_] is much higher than the protein concentration [P_t_] (Arai et al. 2012; Wang et al. 1996; Williamson 2013):

2 $$
 \Delta \delta _{{obs}}  = \Delta \delta _{{\max }} \left\{ {\left( {K_{D}  + [P_{t} ] + [L_{t} ]} \right) - \sqrt {\left( {K_{D}  + [P_{t} ] + [L_{t} ]} \right)^{2}  - 4[P_{t} ]} [L_{t} ]} \right\}/2[P_{t} ] $$

3 $${\Delta\updelta }_{\text{obs}}=\frac{{[\text{L}}_{\text{t}}]}{{\text{K}}_{\text{D}}+{[\text{L}}_{\text{t}}]}{\Delta\updelta }_{\text{max}}$$

where Δδ_obs_ and Δδ_max_ are the observed and maximal change in the chemical shift and K_D_ is the dissociation constant. The error in K_D_ was estimated by manually adjusting K_D_ to higher and lower values and observing the range over which the fit of the CSP data to the model remained within the peak picking error bars of the CSPs.

The fractional populations of the free and bound states were calculated using the peak height (*I*) as follows:

4 $$f_{free} = \frac{{I_{free} }}{{I_{free} + I_{bound} }},\,f_{bound} = \frac{{I_{bound} }}{{I_{free} + I_{bound} }}$$

Here, *f*_*free*_ and *f*_*bound*_ represent the fractions of the protein in the free and bound states. The uncertainties in the peak heights represent the noise level in the spectra. The dissociation constant K_D_ was determined using:

5 $${K}_{D}=\frac{{f}_{free}.\left[{L}_{free}\right]}{{f}_{bound}}$$

In this equation, [*L*_*free*_] represents the concentration of the free ligand, which was approximated as the total ligand concentration [*L*_*total*_] because the ligand was in large excess relative to the protein. The mean K_D_ value was determined by averaging K_D_ values across the used residues.

## Results

To study the interactions between the paramagnetically labeled X3 and the enzyme, ^15^N-labeled BcX E78Q and BcX E78Q-Y69A were titrated with Para-X3 and Dia-X3. The mutation E78Q changes the catalytic Glu to a non-active Gln residue, thus preventing the cleavage of X3. The double mutant BcX E78Q-Y69A has a widened active site, as discussed in detail in our previous paper (Saberi et al. 2024), and was used for reasons explained below. The results for both mutants are presented in parallel.

### TEMPO-labeled xylotriose binds at the SBS

**Fig. 2:**
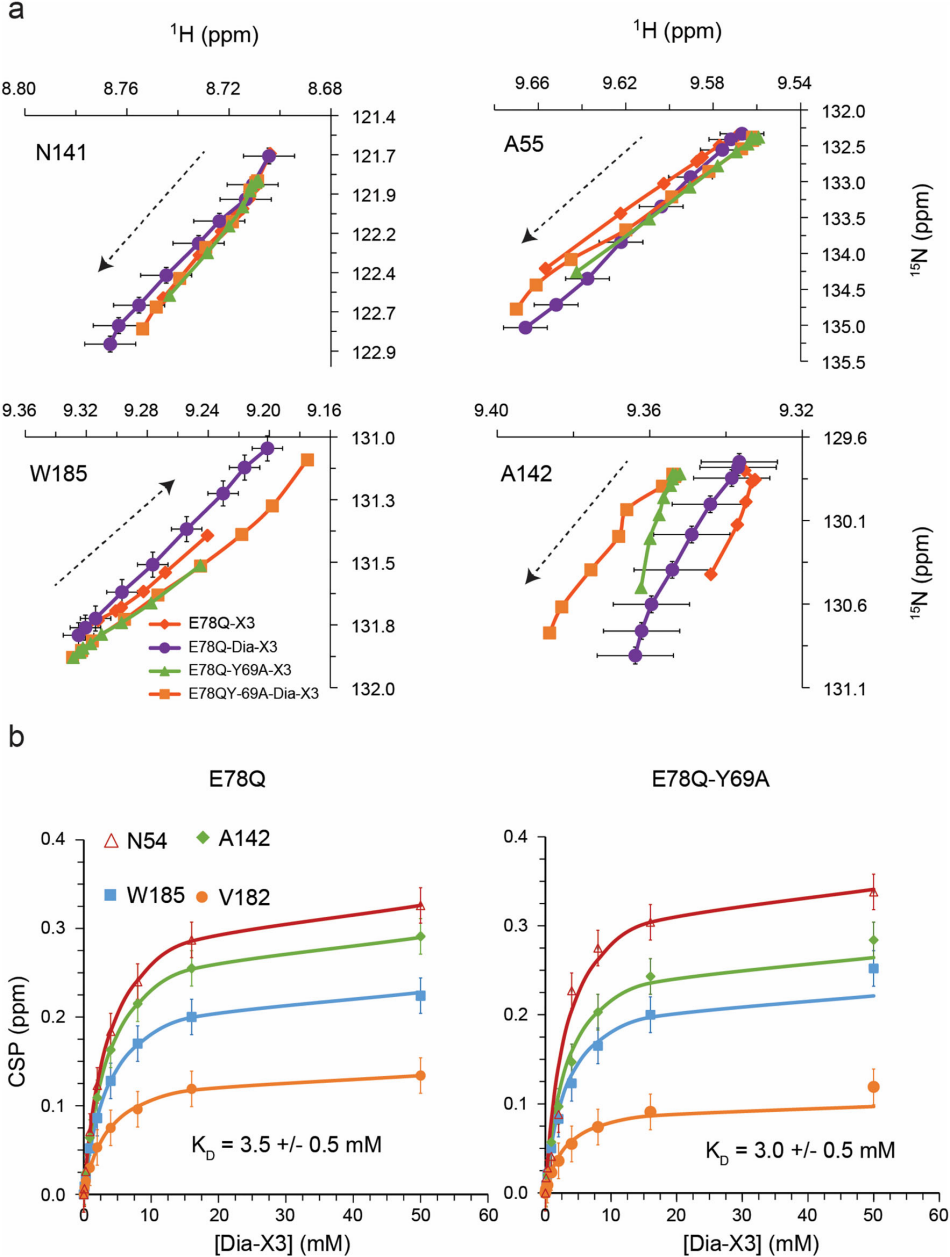
Binding to the SBS. **a** CSP trajectories of SBS amide resonances for BcX E78Q and E78Q-Y69A upon titration with X3 and Dia-X3. Data points are shown with an estimated peak picking error of ± 0.01 ppm for the ^1^H dimension and ± 0.05 ppm for the ^15^N dimension. For clarity, error bars are only shown for the data of BcX E78Q with Dia-X3. **b** Average CSP (Eq. 1) for amide resonances of residues in the SBS of BcX E78Q and BcX E78Q-Y69A are plotted (symbols) against the concentration Dia-X3. K_D_ values were obtained by global fitting (lines) to Eq. 2. Data points are shown with an estimated peak picking error of ± 0.02 ppm. Error in K_D_ is the error of global fitting

The binding of Dia-X3 to ^15^N-labeled BcX E78Q and BcX E78Q-Y69A was studied using NMR spectroscopy to compare the interactions with those observed before for X3 (Saberi et al. 2024). Upon titration with Dia-X3, amide resonances of residues composing the SBS, including N54, A55, N141, T143, N181, T183, and W185, exhibited mainchain and sidechain amide chemical shift perturbations (CSPs) in the fast exchange regime, in line with previous reports and findings for X3 (Ben Bdira et al. 2020; Ludwiczek et al. 2007; Saberi et al. 2024). The similarity in amide trajectory patterns for amides in the SBS during titrations with X3 and Dia-X3 (Fig. 2a) indicates that the two have similar interactions. Dissociation constants (K_D_) of 3.5 ± 0.5 mM for BcX E78Q and 3 ± 0.5 mM for BcX E78Q-Y69A were obtained using a 1:1 binding model (Eq. 2). The data are presented in Fig. 2 and Table 1. The affinity for Dia-X3 to the SBS is higher than found for X3 (23 ± 2 mM), and closer to what was found for X4 (2.3 ± 0.2 mM) (Saberi et al. 2024), suggesting that the TEMPO ring contributes positively to the interaction.

**Table 1:** Dissociation constants (mM) of BcX variants with Para-X3 and Dia-X3 obtained by fitting the CSP curves for amides in the active site, SBS and helix

BcX variant	Active site			SBS			Helix	
	X3^a^	Dia-X3	Para-X3	X3^a^	X4^a^	Dia-X3	Dia-X3	Para-X3
E78Q	5.7(0.2)	3.3(0.4)^b^	NB	23(2)	2.3(0.2)	3.5(0.5)	4.3(0.7)	1.6(0.4)
E78Q-Y69A	K_D1_ = 15(3) K_D2_ = 24(10)	12(2)	NB	22(2)	ND	3.0(0.5)	3.3(0.7)	2.4(0.6)

^a^Taken from (Saberi et al. 2024)

^b^The K_D_ value was determined using the intensity changes from free and bound peaks

Error in K_D_ in brackets is the error over global fitting for residues indicated in the figures

*NB* no binding, *ND* not determined

**Fig. 3:**
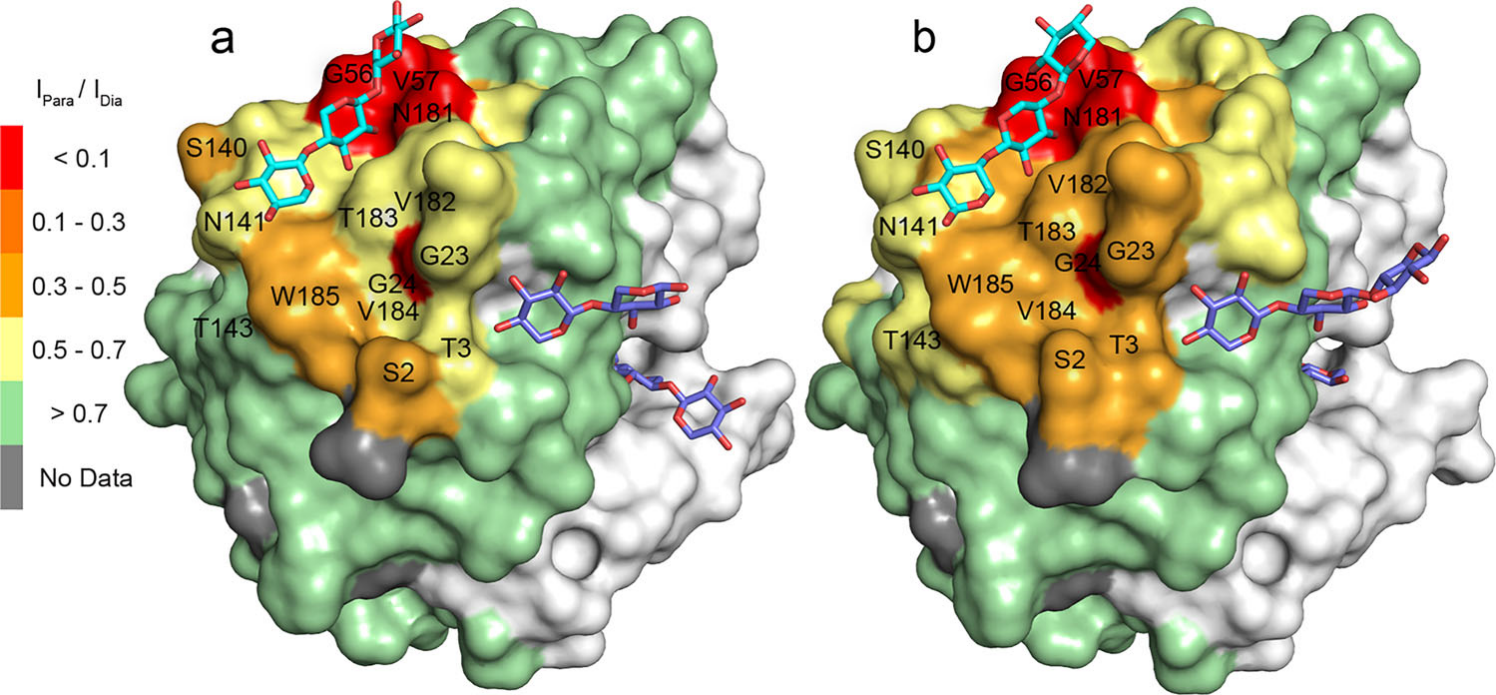
PREs in SBS region. **a**, **b** The peak intensity ratios (*I*_Para_/*I*_Dia_) of SBS amide resonance in the presence of 0.9 mM Para-X3 or 0.9 mM Dia-X3 are color-coded on the structures of BcX E78Q (PDB 8QXY) (**a**) and E78Q-Y69A (PDB 8QY0) (**b**), represented as surfaces. The X3 molecule on the SBS is depicted in cyan sticks, the others in purple sticks. For clarity, PREs are only shown for the SBS region

Addition of 0.9 mM Para-X3 to BcX E78Q or E78Q-Y69A causes the main chain amide resonances of G56, V57 and N181, as well as the sidechain resonances of W185, N181, N141, N25 and N54 to be broadened beyond detection. Also, amide resonances of many other SBS residues show broadening due to PRE. Figure 3 presents PRE maps on the crystal structures of X3 bound BcX variants (PDB 8QXY and 8QY0). PREs affect the entire SBS, with the strongest effects for residues G56, V57, W58, N181, which are close to the non-reducing end of the X3 molecule observed in the crystal, whereas the TEMPO is attached to the reducing end of X3. However, the crystal structure data are not of sufficient quality to determine the orientation of the X3 molecule at the SBS with certainty. The PRE data suggest that Para-X3 predominantly binds with the TEMPO and, thus, also the reducing end of X3, toward the patch formed by G56, V57, W58, N181 and V182. PREs around residues G23 and G24 could also result from interactions of Para-X3 on the side of the SBS, where in the crystal structure another X3 molecule is found.

### Dia-X3 and Para-X3 bind differently to the active site cleft

**Fig. 4:**
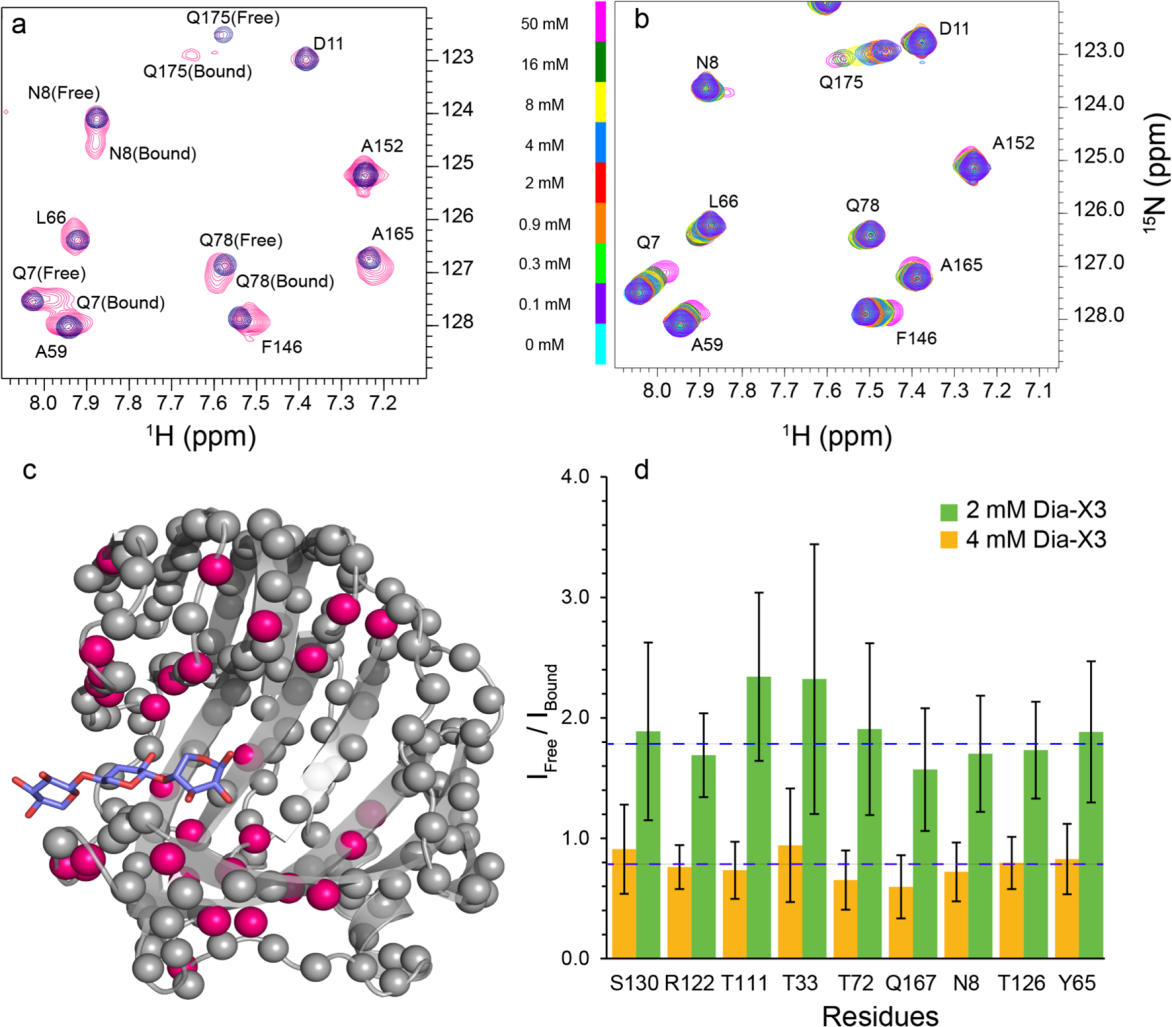
Binding of Dia-X3 inside the active site cleft of BcX. **a**, **b** Detail of overlaid ^1^H − ^15^N HSQC spectra of BcX E78Q in the absence (*blue*) and presence of 2 mM Dia-X3 (*pink*) (**a**) and BcX E78Q-Y69A titrated with Dia-X3 (**b**). The ligand concentrations are indicated next to the color bar. In all samples, the protein concentration was 100 μM. **c** Structure of BcX E78Q in complex with xylotriose (purple sticks) (PDB 8QXY, (Saberi et al. 2024)), in which residues demonstrating peak doubling in titration with Dia-X3 are highlighted with pink spheres. **d** Bar graph displaying the ratios of the free and bound states at 2 and 4 mM. The navy dotted lines indicate the average of intensity ratios. Error bars represent the error from the noise level (1 SD) propagated to intensity ratios

Titration of BcX E78Q with X3 has been shown to result in widespread CSPs across the entire active site cleft, and disappearance of some peaks, indicating fast-to-intermediate exchange on the NMR time scale (Ben Bdira et al. 2020; Ludwiczek et al. 2007). Curved trajectories were observed for amide resonances of some residues, indicating that next to the free protein, at least two other states in fast exchange are present (Saberi et al. 2024). Interestingly, titration of BcX E78Q with Dia-X3 yields resonances in the active site displaying slow-exchange behavior for the free and bound states (Fig. 4, panel a). The binding map shows that the resonances of amides throughout a large part of the active site are affected. The dissociation constant for the binding of Dia-X3 to the BcX active site was estimated based on the resonance intensities of the free and bound states of well-resolved peaks (see examples in Fig. S4) at ligand concentrations of 2 and 4 mM, yielding a K_D_ of 3.3 (0.4) mM (Fig. 4, panel d).

**Fig. 5:**
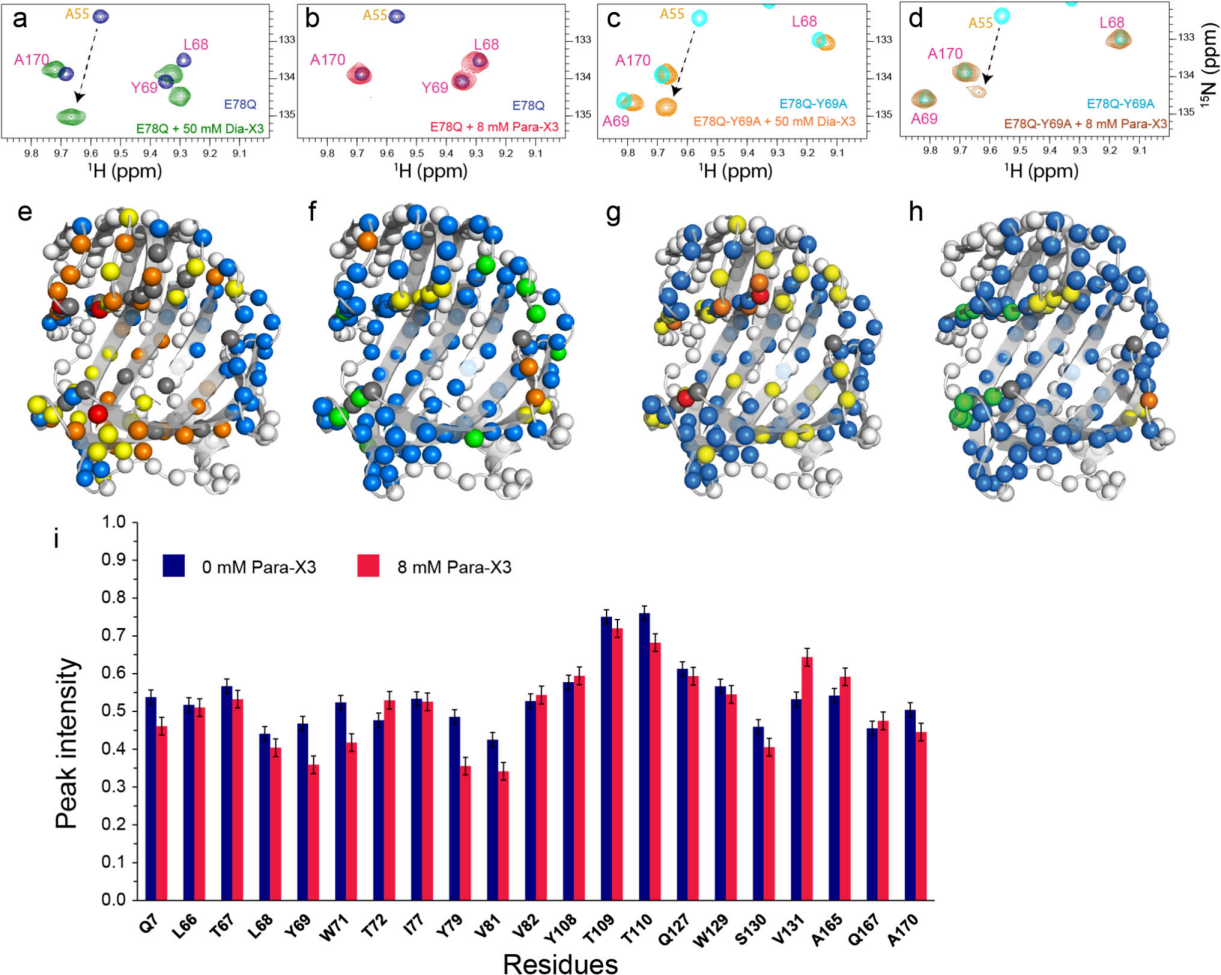
Binding of Para-X3. Top panels: Detail of overlaid ^1^H-^15^N HSQC spectra of BcX E78Q (navy blue) or BcX E78Q-Y69A (cyan), with addition of 50 mM Dia-X3 shown in green (**a**) or orange (**c**) or 8 mM Para-X3 in red-pink (**b**) and brown (**d**). The protein concentration was 100 µM. Residues are indicated with color-coded labels: pink for active site residues, yellow for the SBS. Middle panels: The CSPs of BcX E78Q and BcX E78Q-Y69A in the presence of 50 mM Dia-X3 (**e**, **g**) and 8 mM Para-X3 (**f**, **h**) are color-coded on the backbone amide nitrogen atoms, represented as spheres: blue, CSP < 0.05 ppm; yellow, 0.05 ppm < CSP < 0.1 ppm; orange, 0.1 ppm < CSP < 0.25 ppm; red, CSP > 0.25 ppm; green, resonances disappeared due to PRE effect; grey, no data. For clarity, CSP are only shown for the active site region. Lower panel: **i** Normalized ^1^H-^15^N HSQC peak intensities for amides located inside the BcX active site cleft in the absence (navy blue bars) and presence (red-pink bars) of 8 mM Para-X3, showing the lack of PREs inside the cleft. Peak intensities are normalized to the maximum peak intensity observed in each spectrum across both conditions. Error bars represent the normalized spectral noise level (1 SD)

**Fig. 6:**
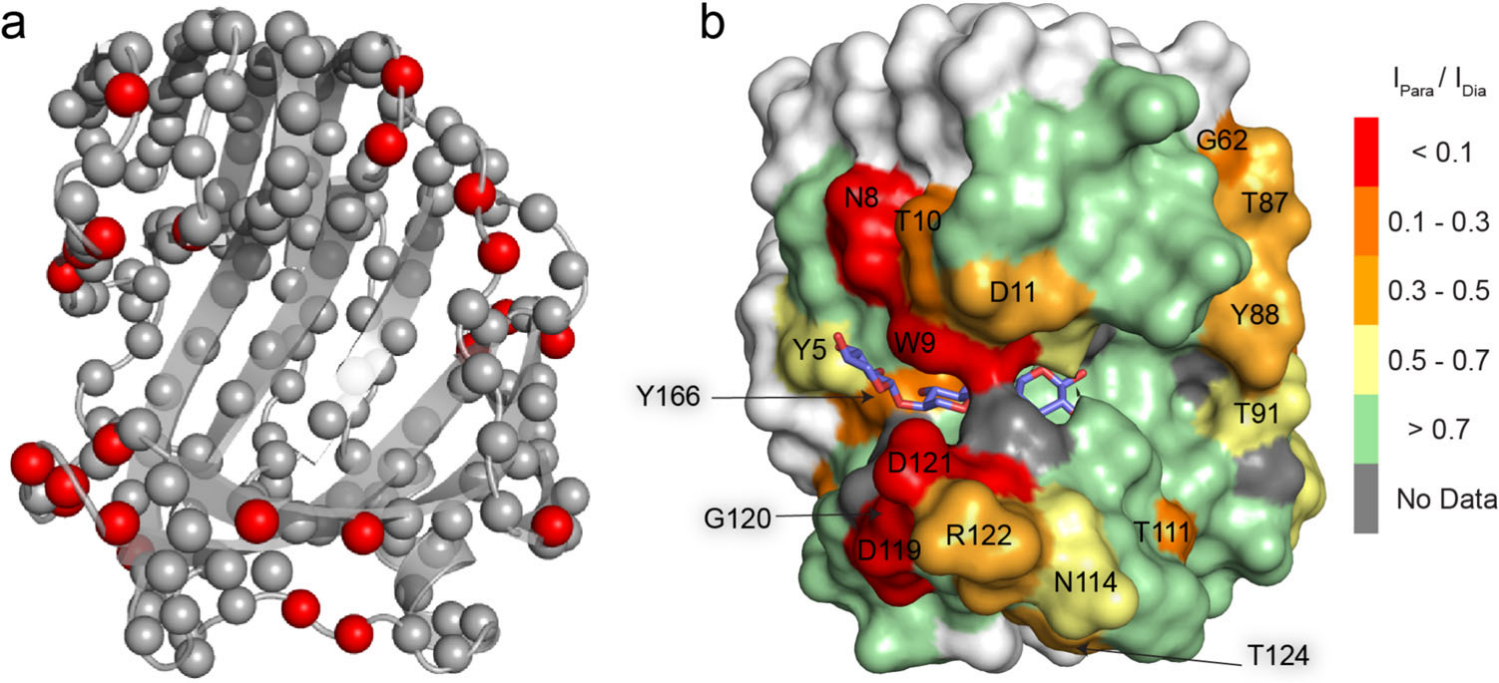
Surface PREs. **a** Amides around the active site cleft that experience PRE in presence of 8 mM Para-X3 are highlighted with red spheres for backbone nitrogen atoms in the structure of BcX E78Q [PDB 8QXY, (Saberi et al. 2024)]; **b** The I_para_/I_dia_ ratios in the presence of 8 mM Para-X3 or 8 mM Dia-X3 are color-coded on the structure of E78Q-Y69A [PDB 8QY0, (Saberi et al. 2024)] shown as a surface model

Addition of Para-X3 does not result in CSP or intensity loss of amide resonances inside the active site cleft (Fig. 5, for more detailed spectra refer to Fig. S5), indicating that binding in the active site cavity is absent or very weak. This contrasts with the results for the diamagnetic control, Dia-X3, which showed slow-exchange binding. To ensure the accuracy of this observation and rule out any potential effects of sample preparation differences, the same 2 mM Para-X3 sample was reduced with sodium ascorbate to generate the corresponding Dia-X3 sample. The resulting spectrum, recorded from the reduced sample, matched that of 2 mM Dia-X3, where slow-exchange binding was observed (Fig. S6). These observations are remarkable, given the minor structural difference between Dia-X3 and Para-X3. PREs are observed outside the cavity of BcX, suggesting that the ligand has transient interactions with several sites on the surface (Fig. 6).

We wondered whether the TEMPO group linked to X3 hindered binding to the active site due to steric restraints, and to test this idea, a variant with an enlarged active cavity was selected. Tyr69 is a highly conserved residue in the − 2 subsite, which is critical for activity and forms hydrogen bonds with the substrate, stabilizing the boat conformation of the xylose ring (Soliman et al. 2009; Wu et al. 2020). Substitution with Ala enlarges the active site due to the loss of the phenol group, causing a lower affinity for X3 in the glycon region (Saberi et al. 2024). Upon titration of BcX E78Q-Y69A with Dia-X3, resonances of active site amides exhibited CSPs in fast exchange on the NMR time scale (Fig. 4, panel b), contrary to the slow exchange observed for BcX E78Q. A dissociation constant of 12 ± 2 mM was obtained. For X3, K_D1_ = 15 (3) mM and K_D2_ = 24 (10) mM had been obtained for binding at glycon and aglycon subsites, respectively (Table 1) (Saberi et al. 2024). Addition of 50 mM Dia-X3 induced CSPs throughout the entire active site cleft with reduced CSPs in the thumb and lower palm region, compared to the BcX E78Q titration with Dia-X3 (Fig. 5, panels e and g). The interactions of Para-X3 (8 mM) with BcX E78Q-Y69A were similar to those with BcX E78Q. No significant CSPs or PRE were observed for amides in the inner palm region (Figs. 5 and 6). Fig. S7 presents detailed ^1^H-^15^N HSQC spectra of E78Q-Y69A in presence of 50 mM Dia-X3 and 8 mM of para-X3. PREs were observed around the entrance of the active site cleft and the -3 subsite, indicating proximity of the TEMPO moiety to this region (Fig. 6).

### Dia-X3 and Para-X3 interact differently with the helical region

**Fig. 7:**
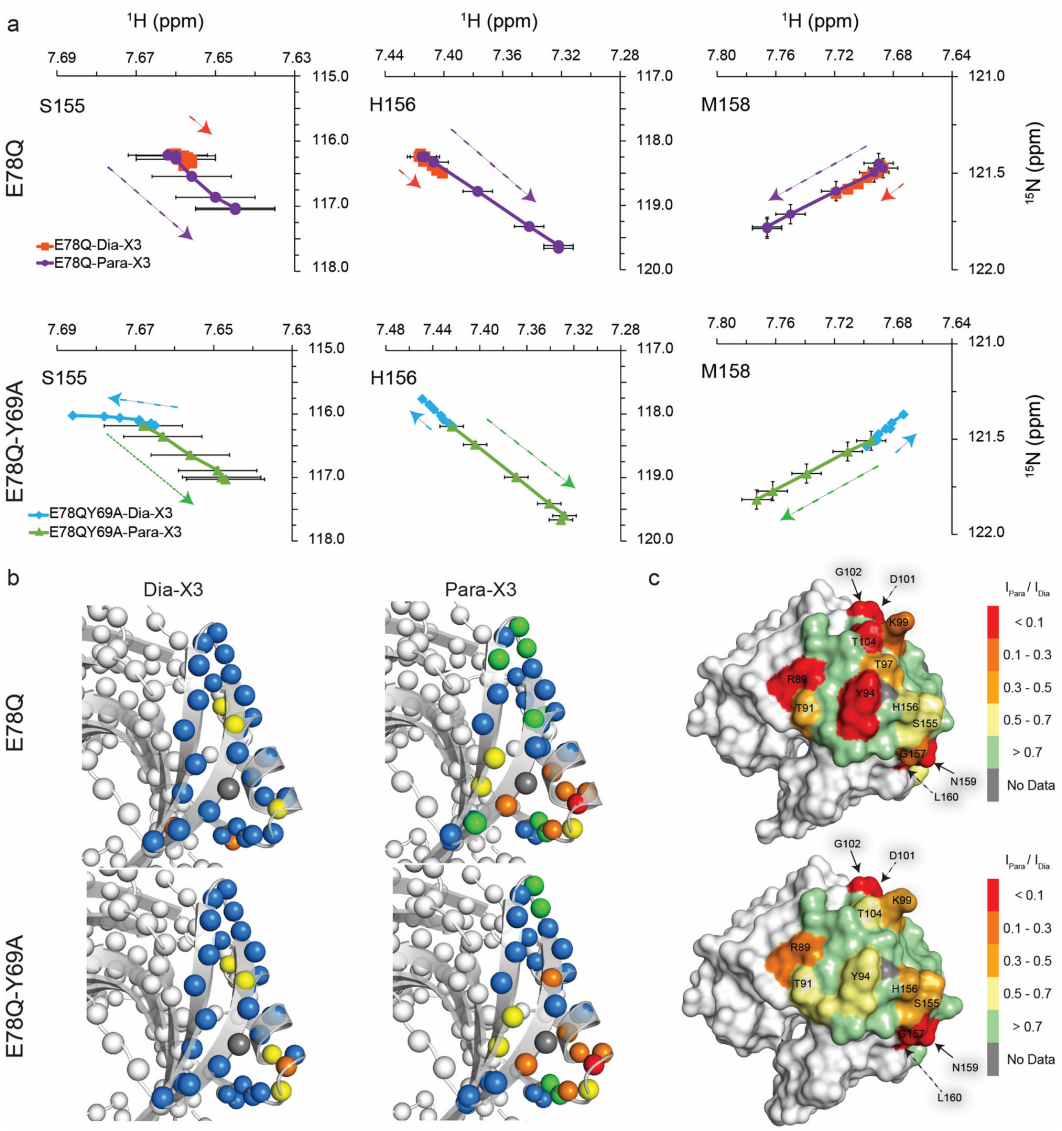
Binding to the α-helical region on BcX. **a** CSP trajectories of α-helical amide resonances for BcX E78Q and E78Q-Y69A upon titration with Dia-X3 and Para-X3. Data points are shown with an estimated peak picking error of ± 0.01 ppm for the ^1^H dimension and ± 0.05 ppm for the ^15^N dimension. **b** The CSPs of BcX E78Q and BcX E78Q-Y69A extrapolated to 100% binding in the presence of Dia-X3 and Para-X3 are color-coded on the backbone amide nitrogen atoms, represented as spheres: blue, CSP < 0.05 ppm; yellow, 0.05 ppm < CSP < 0.1 ppm; orange, 0.1 ppm < CSP < 0.25 ppm; red, CSP > 0.25 ppm; green, resonances disappeared due to PRE effect; grey, no data available. For clarity, CSPs are only shown for the helix region. **c** PREs in the α-helix region. The peak-intensity ratios of amides around the α-helix in the presence of 8 mM Para-X3 or 8 mM Dia-X3 are color-coded on the structures (top) E78Q [PDB 8QXY, (Saberi et al. 2024)] and (bottom) E78Q-Y69A [PDB 8QY0, (Saberi et al. 2024)], both represented as surfaces. For clarity, PREs are only shown for the region surrounding α-helix

In the titrations of BcX E78Q and E78Q-Y69A with Dia-X3 and Para-X3, fast exchange CSPs were observed for amide resonances in the α-helix region. Figure 7 shows the map for Dia-X3 and Para-X3 binding after extrapolation of the CSPs to the value at 100% bound. The affinity for Para-X3 is 2.7 fold (BcX E78Q) and 1.4 fold (BcX E78Q-Y69A) higher than for the diamagnetic equivalent (Table 1 and Fig. S8). For BcX E78Q, the direction of CSPs is the same for Dia-X3 and Para-X3, but curiously, the amplitudes of the CSPs are larger for the latter (Fig. 7). For BcX E78Q-Y69A titrated with Para-X3, the CSP directions and amplitudes are similar as those for BcX E78Q with Para-X3. For Dia-X3, however, the CSPs differ both in amplitude and direction from those observed for BcX E78Q with Dia-X3 (Fig. 7). These observations suggest that Dia-X3 and Para-X3 interact differently with the α-helix region. The larger amplitudes for Para-X3 suggest a more specific interaction with BcX than Dia-X3 has. The PRE map shows that in both mutants, the resonances of residues N159, L160 and G157, located within a short loop connecting α-helix to a β-strand, broaden beyond detection, suggesting that TEMPO interacts with these residues. Additionally, a pronounced PRE effect was observed for K99 and Y94 upon addition of 8 mM Para-X3 in E78Q variant; however, the corresponding residues in E78Q-Y69A exhibit a less prominent PRE effect.

## Discussion

GH11 xylanases exhibit remarkable specificity in their enzymatic action, which is attributed to the unique morphology of their active sites. These enzymes possess a narrow, long, cleft-shaped active site and have a high affinity for unsubstituted consecutive xylose residues (Biely et al. 1997; Davies et al. 1997; Törrönen et al. 1997). The active site tunnel is lined with both polar and bulky aromatic residues, creating an optimal network of hydrogen bond and CH − π interactions for positioning the substrate (Madan et al. 2018; von Schantz et al. 2014). The narrowest part of the active site cleft is located between Trp9 and Pro116 in the middle, and between Tyr88 and Tyr174 at the end of the cleft (Paës et al. 2012, 2007). The enzymes also have an SBS on the surface of the enzyme, which is thought to assist in the processive hydrolysis of glycosidic linkages on the xylan substrate. The similarity of the amide peak trajectories upon titrating with X3 and Dia-X3 suggests that Dia-X3 and X3 bind to the SBS in a similar manner, though the TEMPO group appears to contribute positively to the interactions, because the affinity for Dia-X3 is higher than for X3 itself. The binding of Para-X3 causes extensive PRE, and even at moderate concentration, the amide resonances which are closest to the TEMPO group broaden beyond detection, in line with expectation.

**Fig. 8:**
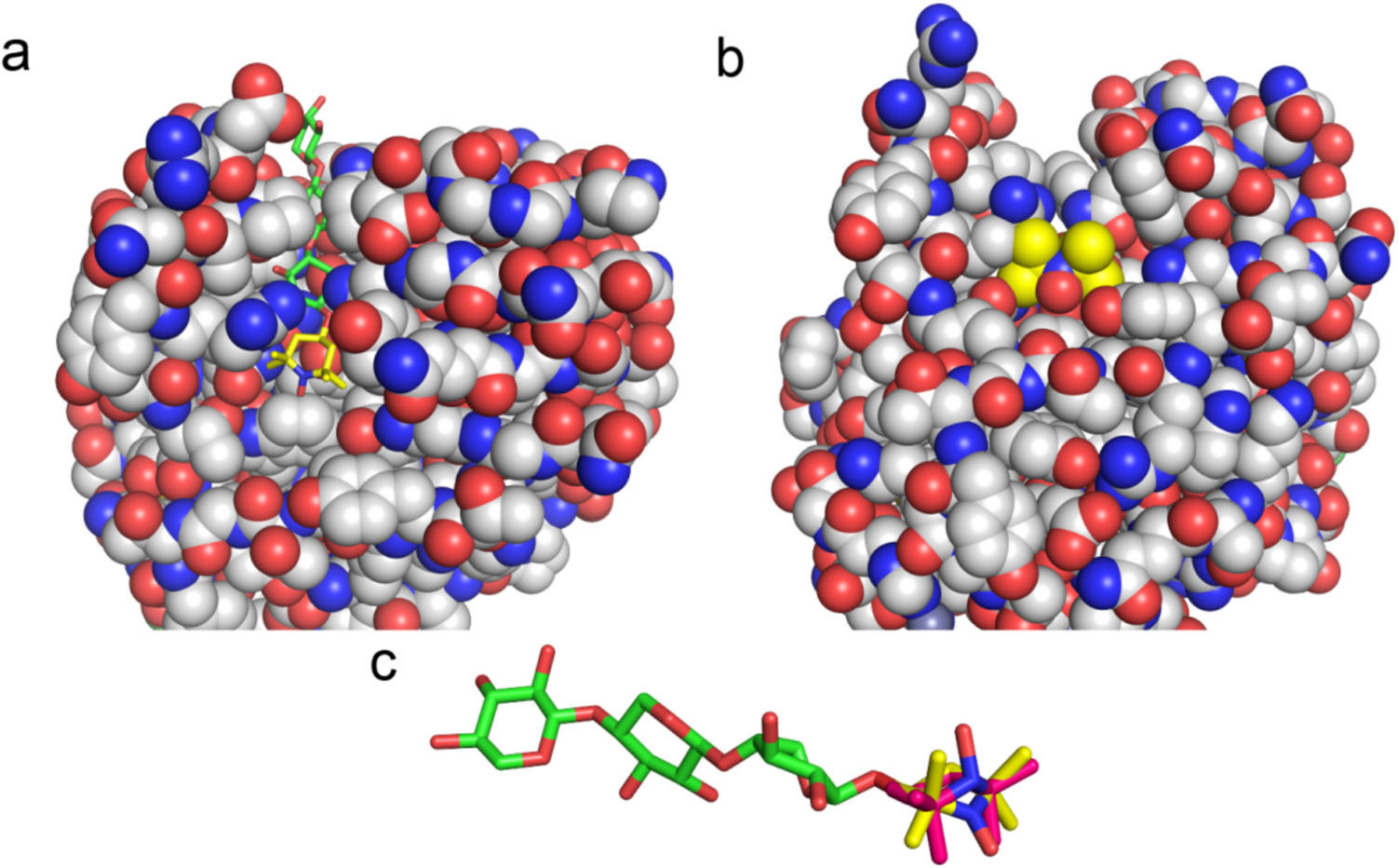
Model of Para-X3 and Dia-X3 in BcX. **a, b** TEMPO [sp^2^ configuration, yellow, taken from PDB 6V51 (Liu et al. 2020)] was modelled attached to the reducing end of X3 (green) present in the active site of BcX [PDB: 8QXY, (Saberi et al. 2024)]. The protein is shown in spacefill representation with the O, N and C atoms in red, blue and grey. Para-X3 is shown in sticks (**a**) and spacefill (**b**). Note the steric clashes of the methyl groups of TEMPO with the protein. **c** In the same model as in (**a**), a TEMPO in sp^3^ configuration [magenta, PDB 3EFT, (Ciani et al. 2009)] is overlaid with the sp^2^ TEMPO. Note the rearrangement of the six-membered ring

Binding of the TEMPO-labeled X3 in the active site cleft, is, however, different from regular X3. For BcX E78Q, the TEMPO group attached to X3 in the diamagnetic state (Dia-X3) slows the exchange between free and bound in the cleft from the fast-to-intermediate regime to the slow regime (Fig. 4, panel a). Perhaps the TEMPO group causes steric hinderance when entering and leaving the narrow active site, raising the activation barrier of binding. Remarkably, binding of Para-X3 cannot be detected inside the cleft. Only small CSPs and PREs are observed for resonances of amides located outside and around the edges of active site, but not inside the cleft (Figs. 5 and 6). In BcX E78Q-Y69A, removal of the bulky phenol ring of Tyr69 enlarges the active site and lowers the affinity of X3 to the glycon site. For Dia-X3 binding, this mutation brings back the exchange to the fast regime. However, binding of Para-X3 is also not observed for this BcX variant. Upon the addition of 8 mM Para-X3, a notable PRE effect was observed for amides located at the entrance and edges of the active cleft, while the resonances of amides inside the active site cleft exhibited negligible PRE. For the purpose of visualization, we modelled the TEMPO molecule (taken from PDB 6V51, (Liu et al. 2020)) attached to the X3 observed in the active site of BcX in a crystal structure (PDB 8QXY, (Saberi et al. 2024)) as shown in Fig. 8. The model is only approximate because it was not optimized with molecular dynamics, but it helps to show the tight fit of the TEMPO molecule in the active site. The methyl groups clash with atoms of the protein.

The single α-helix located beneath the β-strands contributes to stability (Paës et al. 2012). Molecular dynamics (MD) simulations of the xylanase from *Paenibacillus xylanivorans A57*, PxXyn11B, with X3 suggested the existence of an additional binding site for the substrate in this region (Briganti et al. 2021). Moreover, recent reports have highlighted the identification of an additional substrate-binding site situated near the α-helix, spanning residues F146-S155, and extending to the short loop between residues H156-S162 (Molina et al. 2024). A NMR-monitored titration of BcX-WT with cellopentaose resulted in CSPs for amides (153–157) located in region surrounding the α-helix (Ludwiczek et al. 2007). Titration of BcX E78Q with different xylose oligomers (X2, X3, X4, X5 and X6) did not cause significant CSPs for amides in this region but both Dia-X3 and Para-X3 show interactions and also in this case, differences are observed for the two compounds, as evidenced by the disparities in the directions and amplitudes of CSPs.

The use of nitroxide radicals as probes has a long history in the study of biological macromolecules by NMR and EPR spectroscopy (Griffith et al. 1969; Hustedt et al. 1999; Jahnke 2002; Klare et al. 2009; Kosen 1989; Okuno et al. 2022; Torricella et al. 2021). These radicals are not only used for spin-labeling proteins but also ligands. Johnson et al. used TEMPO-labeled sugars to explore the binding dynamics and spatial orientation of sugar chains within the cellulose-binding domains of *Cellulomonas fimi* β-1,4-glucanase CenC (Johnson et al. 1999). In a similar vein, the utilization of a nitroxide spin-labeled analogue of N-acetyllactosamine with galectin-3, a mammalian lectin, facilitated precise mapping of the oligosaccharide's binding sites on the protein surface (Jain et al. 2001). The reduced form of the nitroxide radical is generally used as the diamagnetic control in such studies and we are not aware of reports showing that the radical and reduced forms result in different effects on the system of interest. Chemical differences between the radical and the reduced forms are small but not absent. The nitrogen atom in the radical form is expected to mainly be in sp^2^ configuration, whereas in the reduced form the sp^3^ configuration is expected. Furthermore, the oxygen binds a proton, changing the charge distribution and hydrogen bonding properties of the NO group (Giffin et al. 2011; Yonekuta et al. 2007). In Fig. 8c, a TEMPO in sp^3^ (tetrahedral) configuration (taken from PDB 3EFT, (Ciani et al. 2009) is overlaid with the TEMPO in sp^2^ (flat) configuration, showing significant rearrangement in the six-membered ring, from ‘boat’ in sp^2^ to ‘chair’ in the sp^3^ configuration. We propose that it must be these changes that are the cause of the remarkable differences found for the behavior of Dia-X3 and Para-X3. This study can serve as a reminder that any probe can affect the behavior of the system of interest and attachment of a paramagnetic group to a small molecule, such as an enzyme substrate, may well be more likely to interfere than probes attached to the surface of proteins.

## Supplementary Information

Below is the link to the electronic supplementary material.

Supplementary file1 (PDF 5934 KB)

## Data Availability

Data is provided within the manuscript or supplementary information files.
