# Supplementary material for: Terminal spin labeling of xylotriose strongly affects interactions in the active site of xylanase BcX
Source: J Biomol NMR. 2025 Mar 12;79(2):99–113. doi: 10.1007/s10858-025-00459-w (PMC12078400; doi:10.1007/s10858-025-00459-w)
Supplement: Supplementary file 1 — Supplementary file1 (PDF 5934 KB) [file 10858_2025_459_MOESM1_ESM.pdf]

Supporting material

Terminal spin labeling of xylotriose strongly affects interactions in the active site of xylanase BcX

Mahin Saberi[1], René Dekkers[1,2], Leonardo Passerini[3], Martina Huber[3], Mark Overhand[2], Marcellus Ubbink[1] *

[1] Department of Macromolecular Biochemistry, Leiden Institute of Chemistry, Leiden University, Einsteinweg 55, 2333 CC Leiden, The Netherlands

[2] Department of Bio-organic Synthesis, Leiden Institute of Chemistry, Leiden University, Einsteinweg 55, 2333 CC Leiden, The Netherlands

[3] Department of Physics, Huygens-Kamerlingh Onnes Laboratory, Leiden University, Niels Bohrweg 2, 2333 CA Leiden, The Netherlands

* Corresponding author, email: m.ubbink@chem.leidenuniv.nl

Supplementary figures

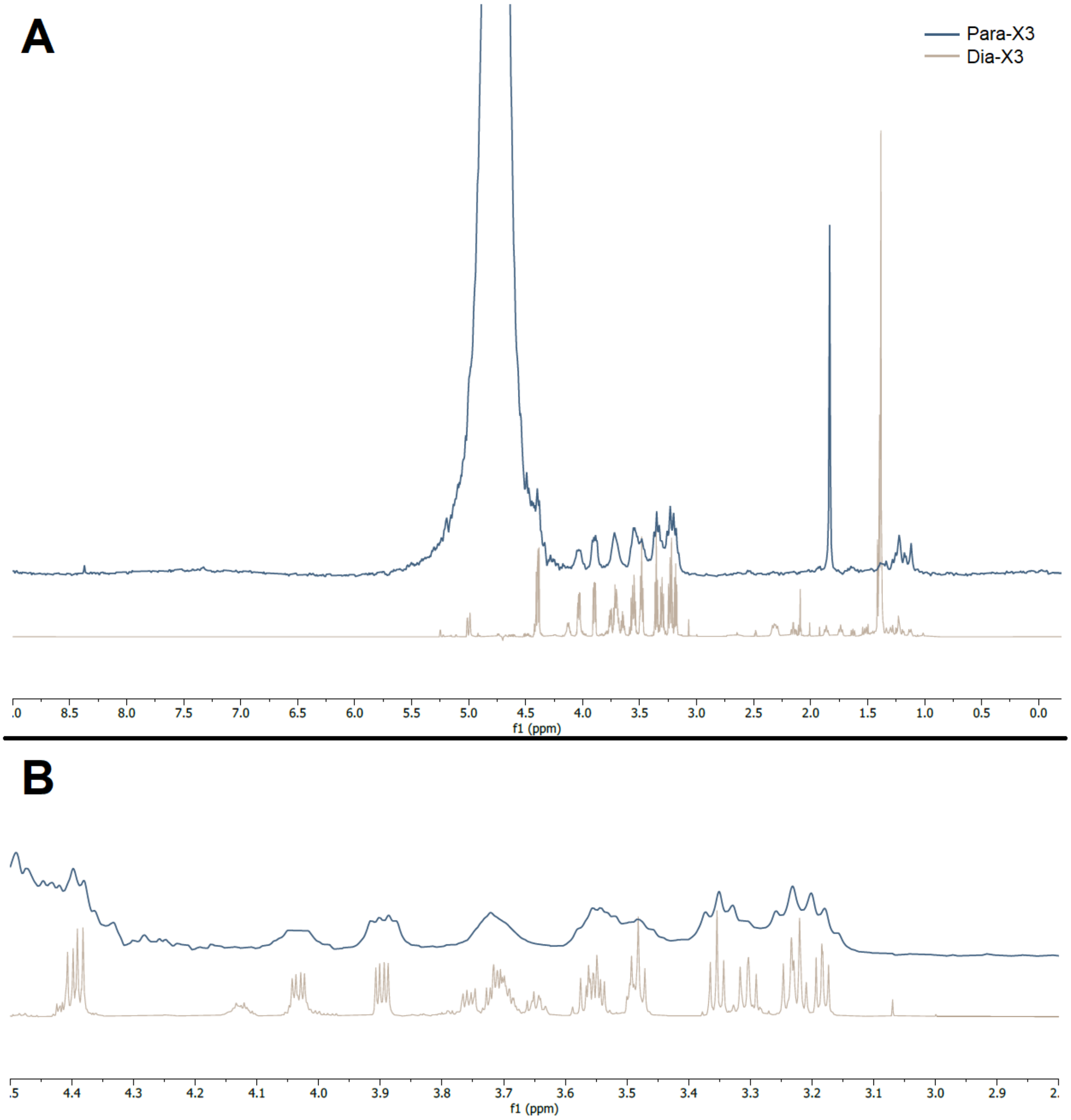

Fig. S1 ^{1}H NMR. (**a**) overlay of Dia-X3 (taupe) with Para-X3 (blue); (**b**) Detail of the 2.8-4.5 ppm range

* Corresponding author, email: m.ubbink@chem.leidenuniv.nl

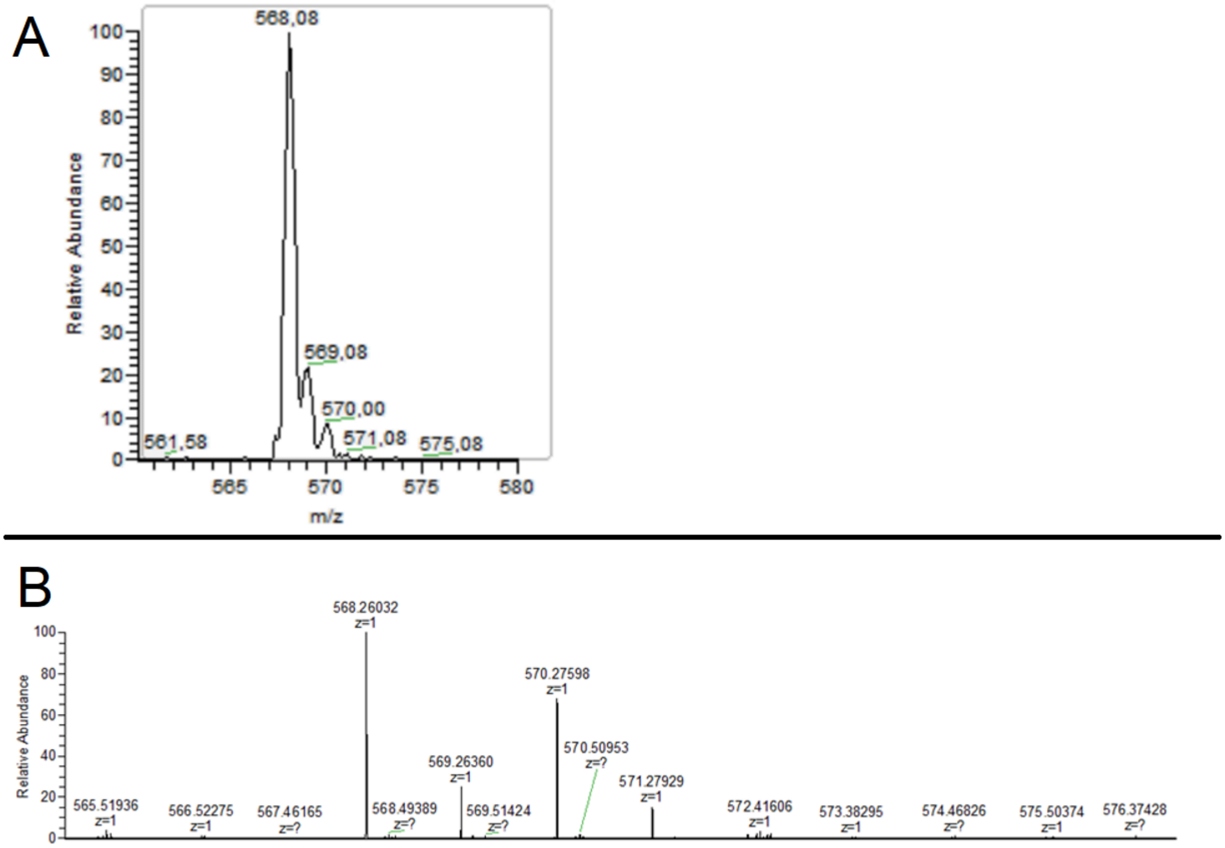

Fig. S2 Mass analysis. (**a**) LC-MS analysis of the paramagnetic substrate shortly before addition to the enzyme. The 568.08 and 569.08 (single ^{13}C atom) correspond to the desired substrate based on the fragmentation mechanism of TEMPOL upon electrospray ionization (ESI) treatment (Smith et al. 2000). Some diamagnetic substrate is present as well (masses 570.00 and 571.08), observed at +2 Da. The paramagnetic fraction is estimated to be 93%; (**b**) HR-MS analysis of Para-X3, ($[C_{24}H_{43}NO_{14}]^+$, 568.260 and single ^{13}C mass 569.264) and Dia-X3,($[C_{24}H_{43}NO_{14} + H]^+$, 570.276 and single ^{13}C mass 571.279)

* Corresponding author, email: m.ubbink@chem.leidenuniv.nl

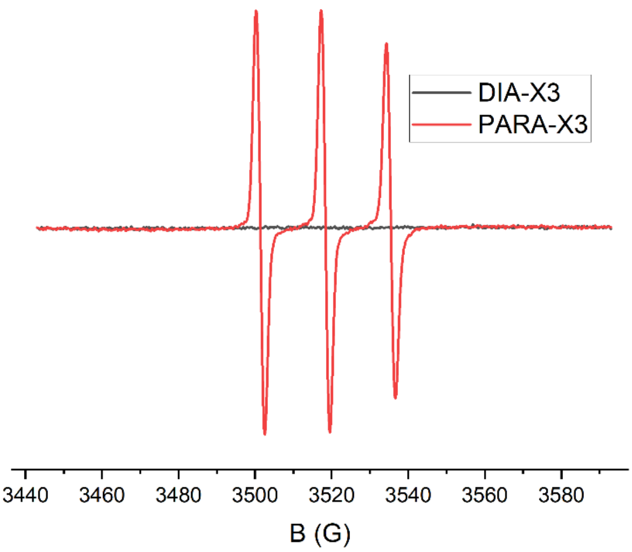

Fig. S3 Room temperature continuous wave EPR spectra of Dia-X3 (black) and Para-X3 (red). The Para-X3 spectrum shows the typical three-line spectrum of a nitroxide radical in fast motion, whereas Dia-X3 is EPR inactive, i.e. diamagnetic. The spectra were acquired with an EMX Plus EPR spectrometer (Bruker BioSpin, Germany) with an SHQ resonator. EPR measurement conditions: microwave frequency 9.88 GHz, modulation frequency: 100 KHz, modulation amplitude: 0.3 mT, time constant: 20.48 ms, power: 10.02 mW, measurement time: two min

* Corresponding author, email: m.ubbink@chem.leidenuniv.nl

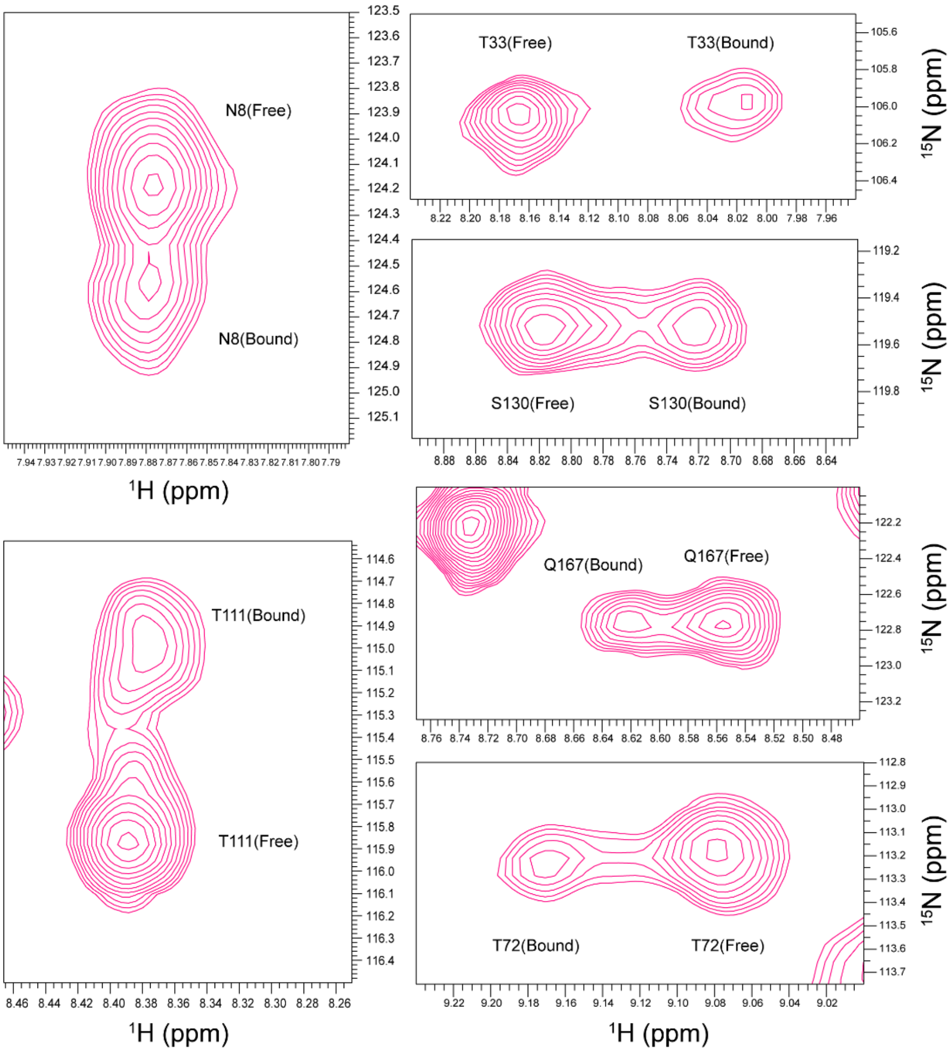

Fig. S4 Details of the ^{1}H–^{15}N HSQC spectrum of BcX E78Q with 2 mM Dia-X3 showing examples of peaks for which the intensities of the free and bound states were used for K_D calculation

* Corresponding author, email: m.ubbink@chem.leidenuniv.nl

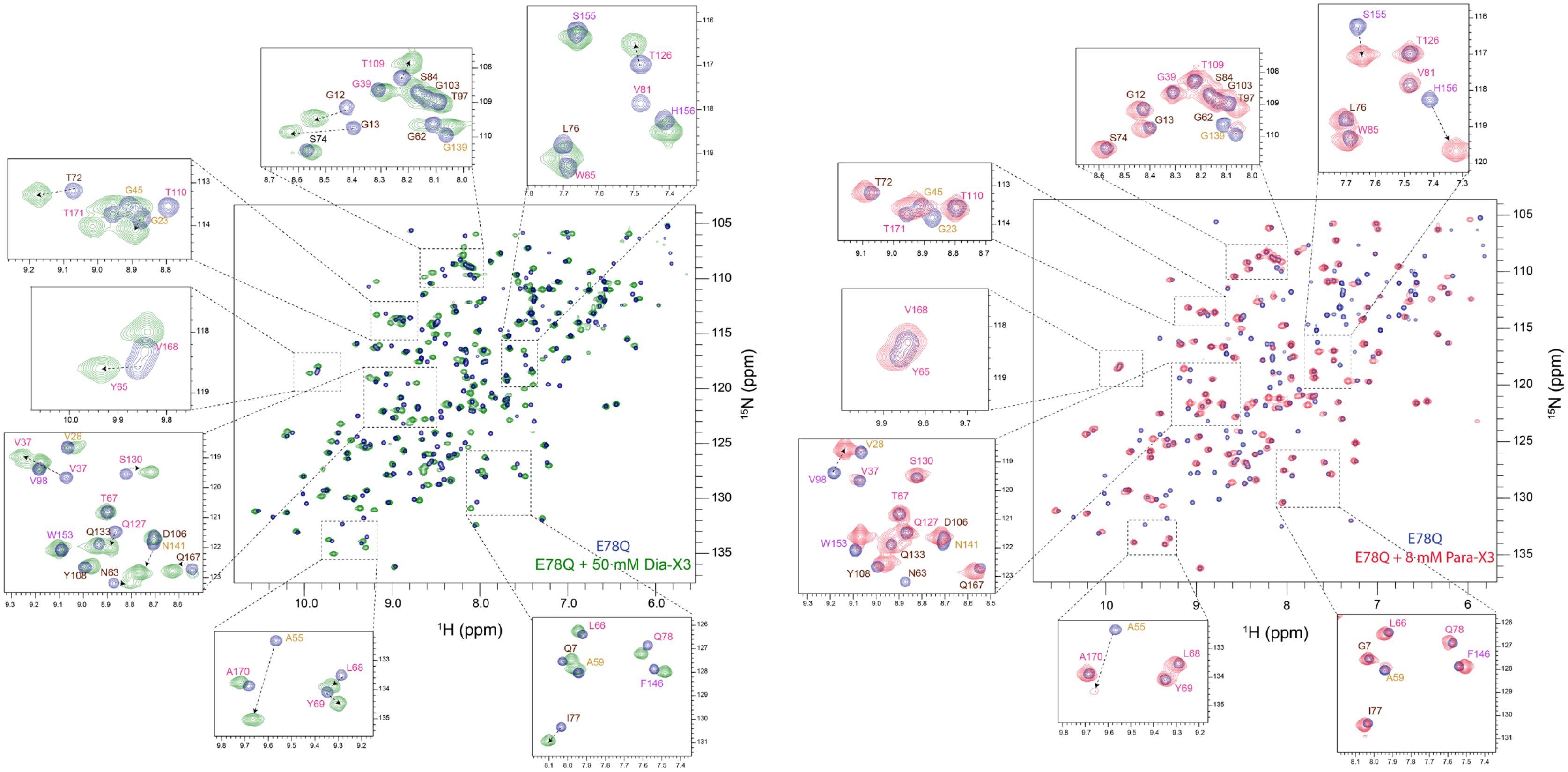

Fig. S5 Differential binding effects. Overlaid ^{1}H-^{15}N HSQC spectra of BcX E78Q (navy blue), with addition of (left) 50 mM Dia-X3 shown in green and (right) 8 mM Para-X3. The protein concentration was 100 µM. Residues are in color-coded labels to indicate specific regions: pink for active site residues, yellow for the SBS, purple for residues surrounding the α-helix region and maroon for residues at the edges of the active site

* Corresponding author, email: m.ubbink@chem.leidenuniv.nl

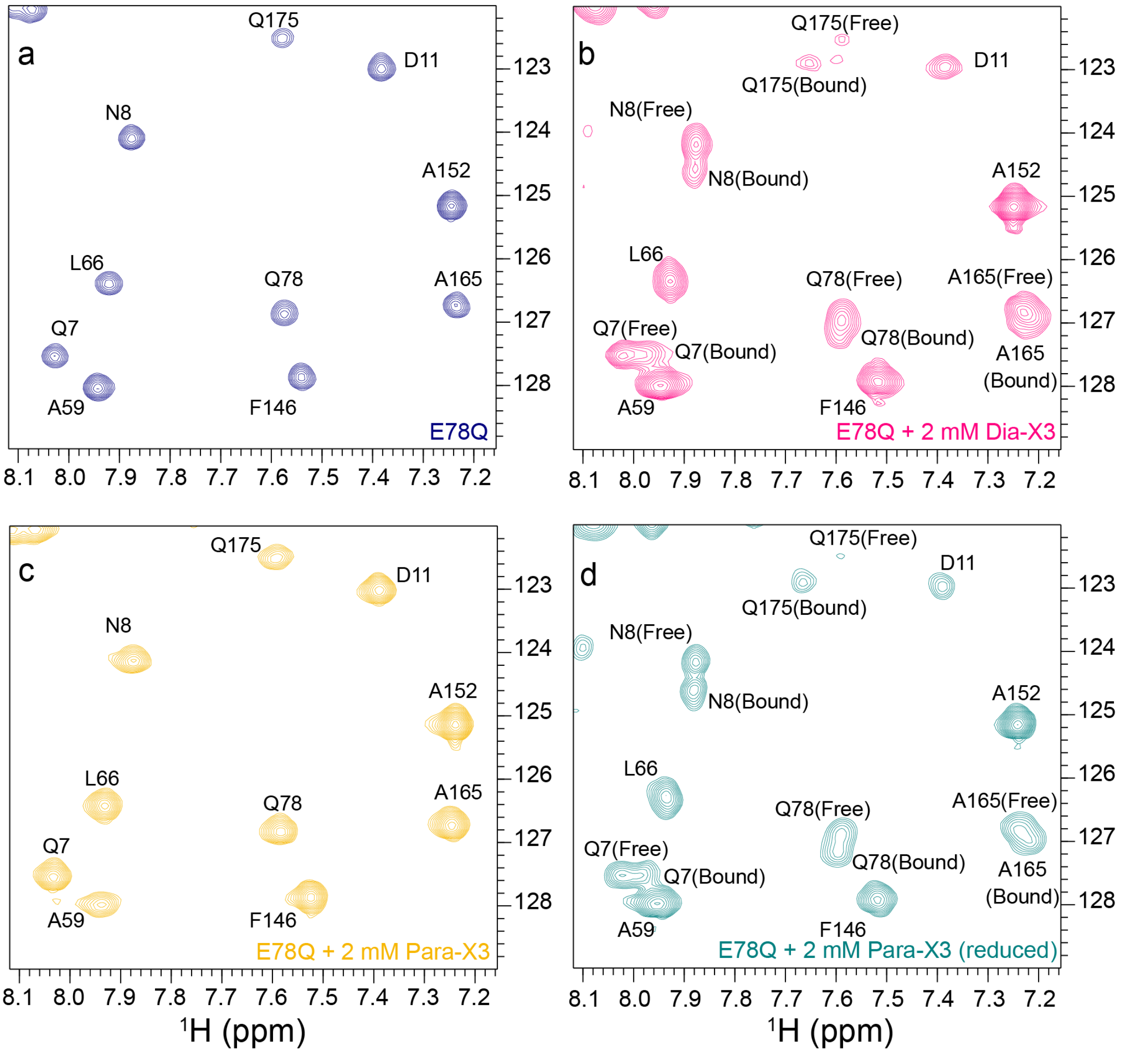

Fig. S6 HSQC spectra of BcX E78Q. Selected regions from ^{1}H–^{15}N HSQC spectra of BcX E78Q under various conditions: (**a**) in free form shown in navy; (**b**) in the presence of 2 mM Dia-X3 in pink; (**c**) in the presence of 2 mM Para-X3 shown in yellow; (**d**) displaying the spectrum after reducing the sample from (**c**) with sodium ascorbate

* Corresponding author, email: m.ubbink@chem.leidenuniv.nl

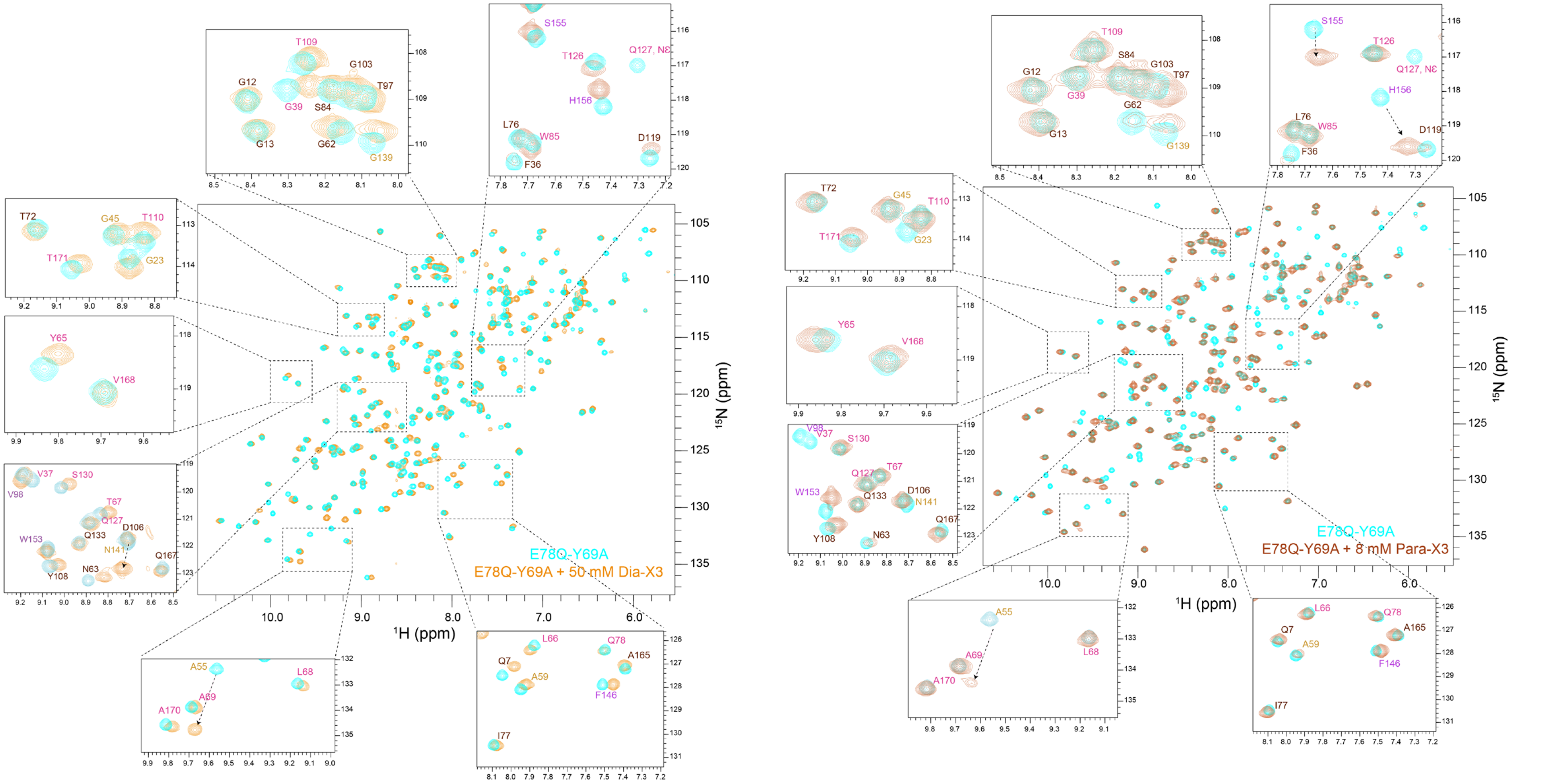

Fig. S7 Interactions with BcX E78Q-Y69A. Overlaid ^{1}H-^{15}N HSQC spectra of BcX E78Q-Y69A depicted in cyan, with addition of (left) 50 mM Dia-X3 shown in orange and (right) 8 mM Para-X3 in brown. The protein concentration was 100 µM. Residues highlighted with color-coded labels to indicate specific regions: pink for active site residues, yellow for the SBS, and purple for residues surrounding the α-helix region and maroon for residues at the edges of the active site

* Corresponding author, email: m.ubbink@chem.leidenuniv.nl

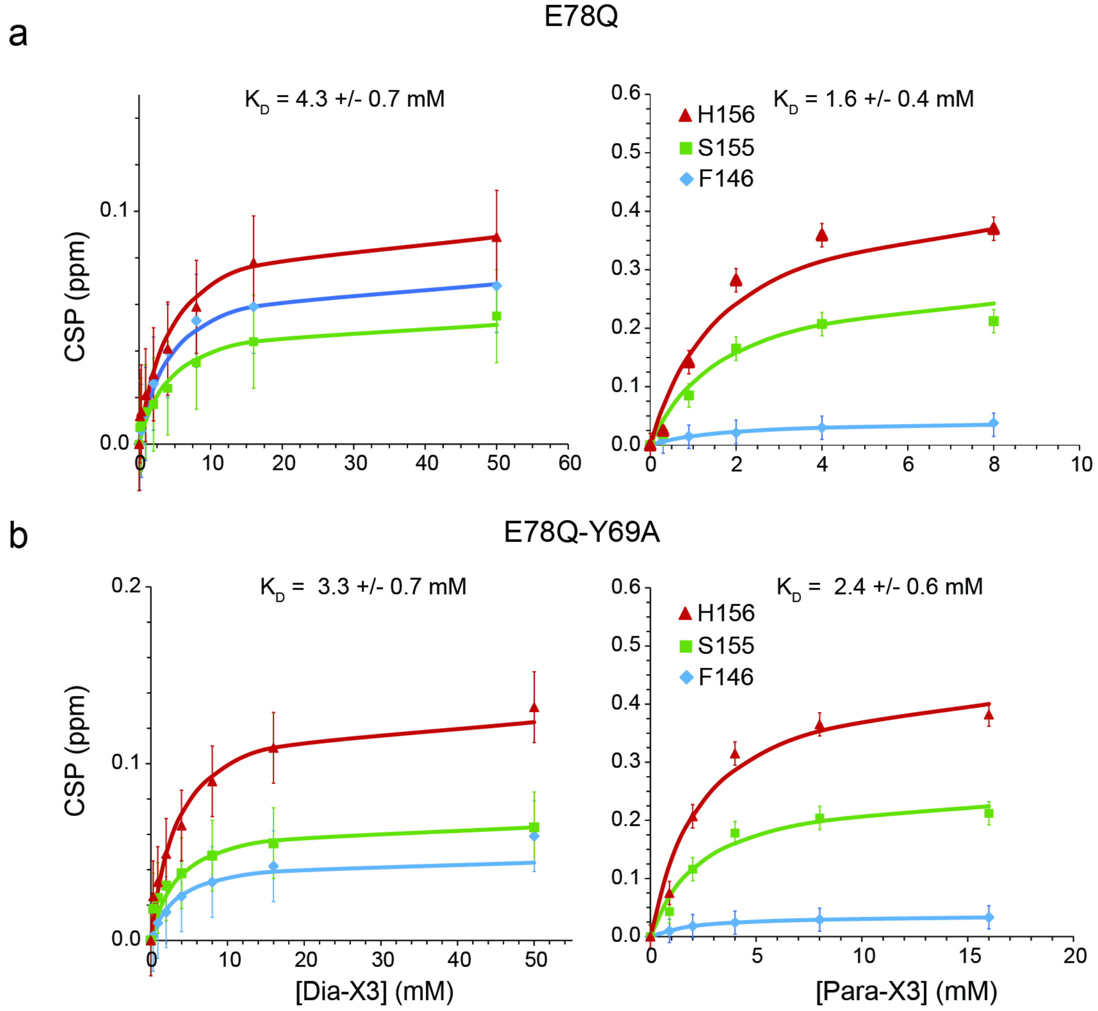

Fig. S8 Binding to the helix region. Average CSP (equation 1) for the indicated amide resonances of residues in the α-helix for (**a**) BcX E78Q and (**b**) BcX E78Q-Y69A are plotted (symbols) against the concentration Dia-X3 (left) or Para-X3 (right). K_D values were obtained by global fitting (lines) to equation 2a. Data points are shown with an estimated peak picking error of ± 0.02 ppm. Error in K_D is the error of global fitting

* Corresponding author, email: m.ubbink@chem.leidenuniv.nl

NMR spectra synthesis

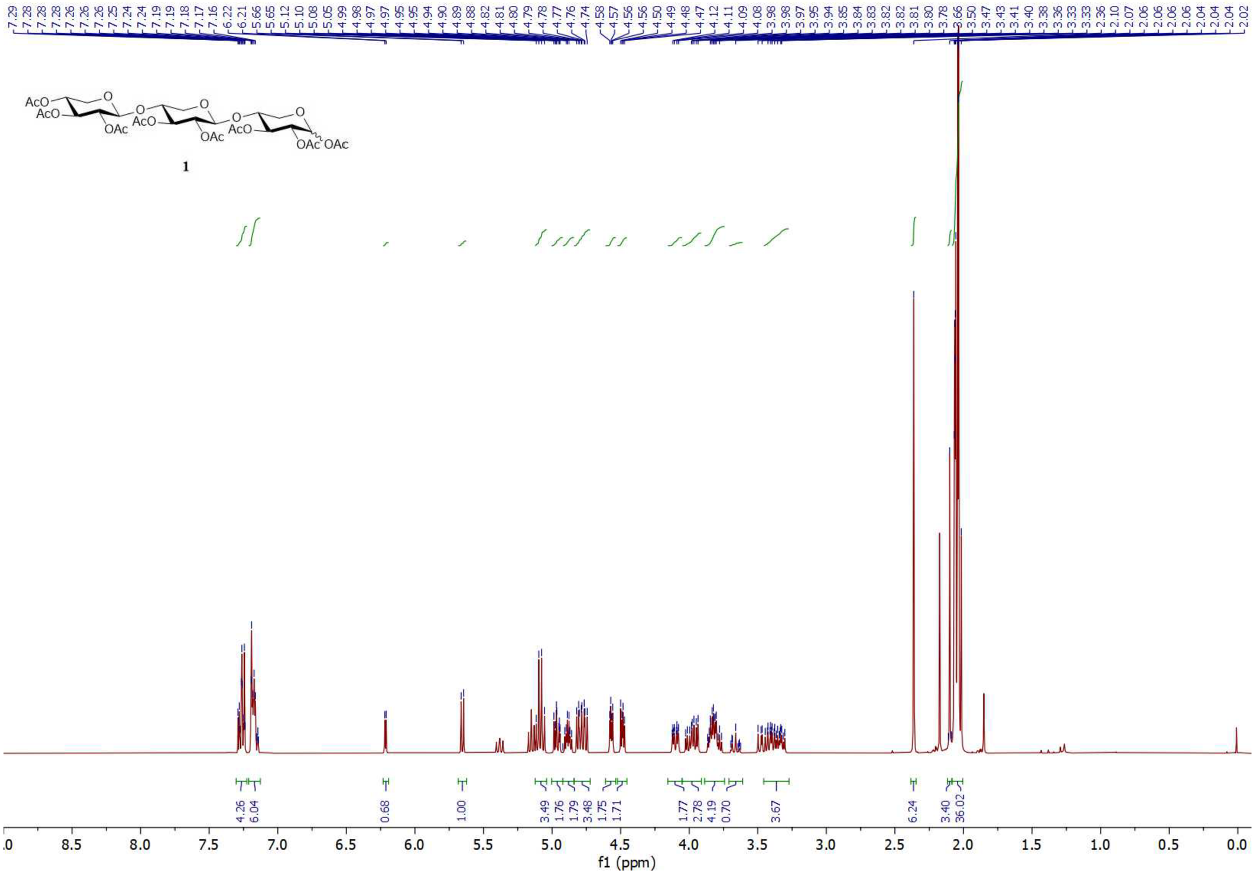

* Corresponding author, email: m.ubbink@chem.leidenuniv.nl

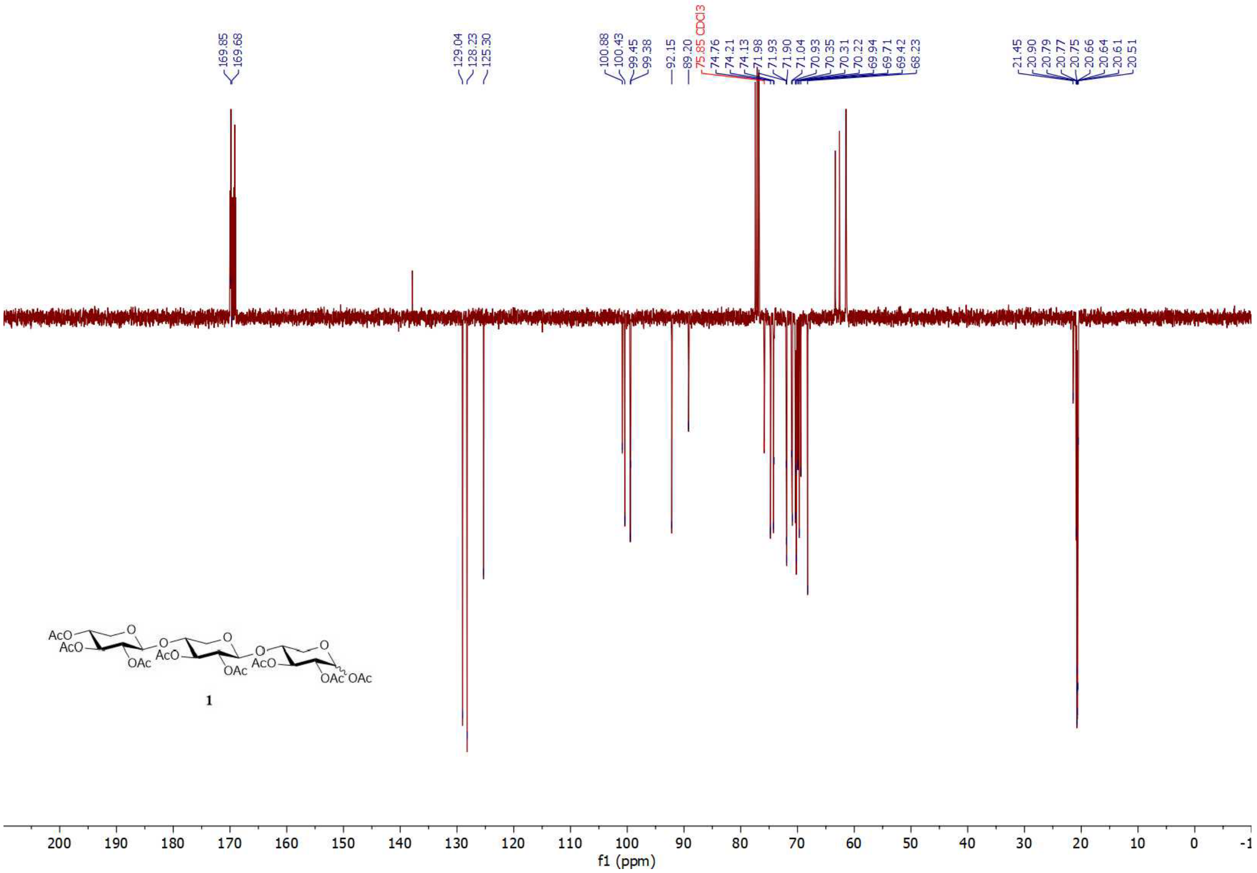

* Corresponding author, email: m.ubbink@chem.leidenuniv.nl

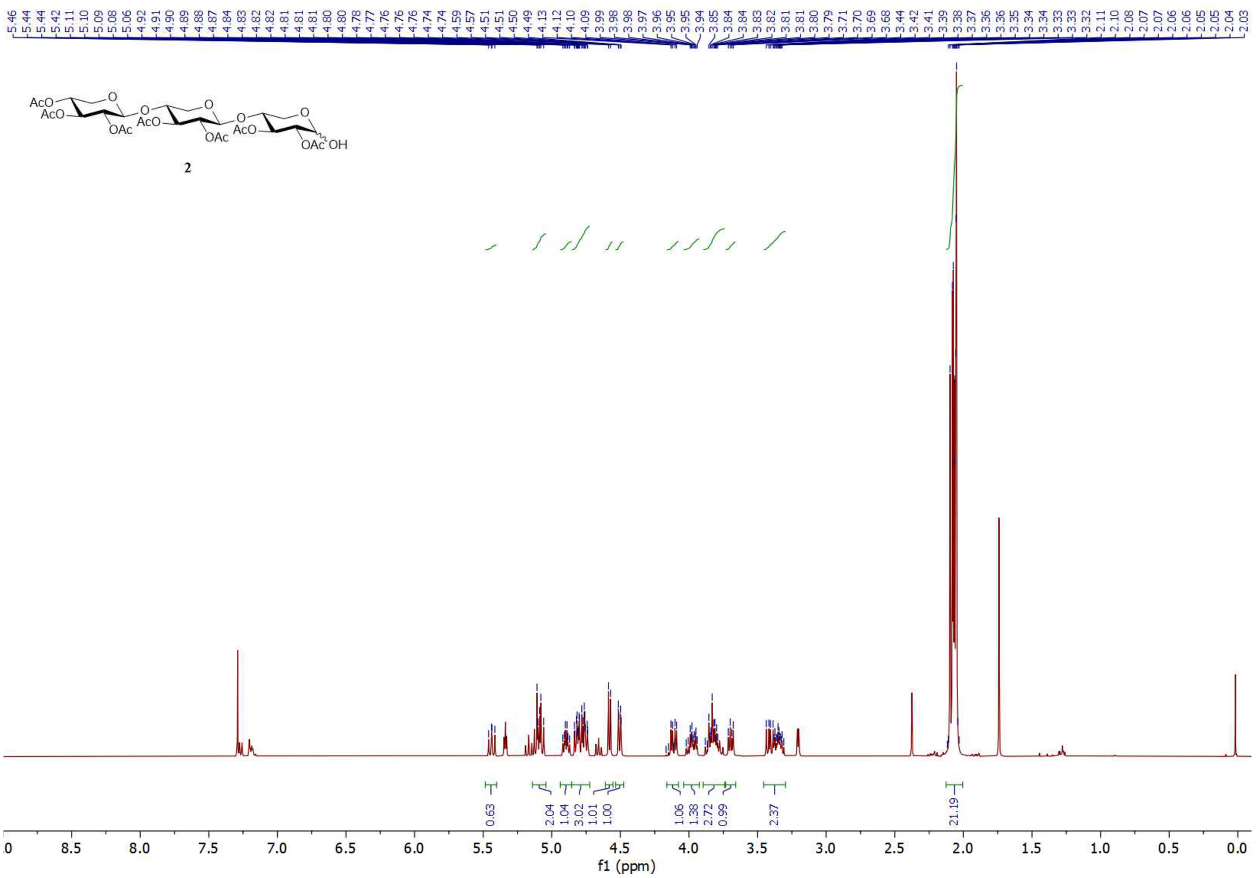

* Corresponding author, email: m.ubbink@chem.leidenuniv.nl

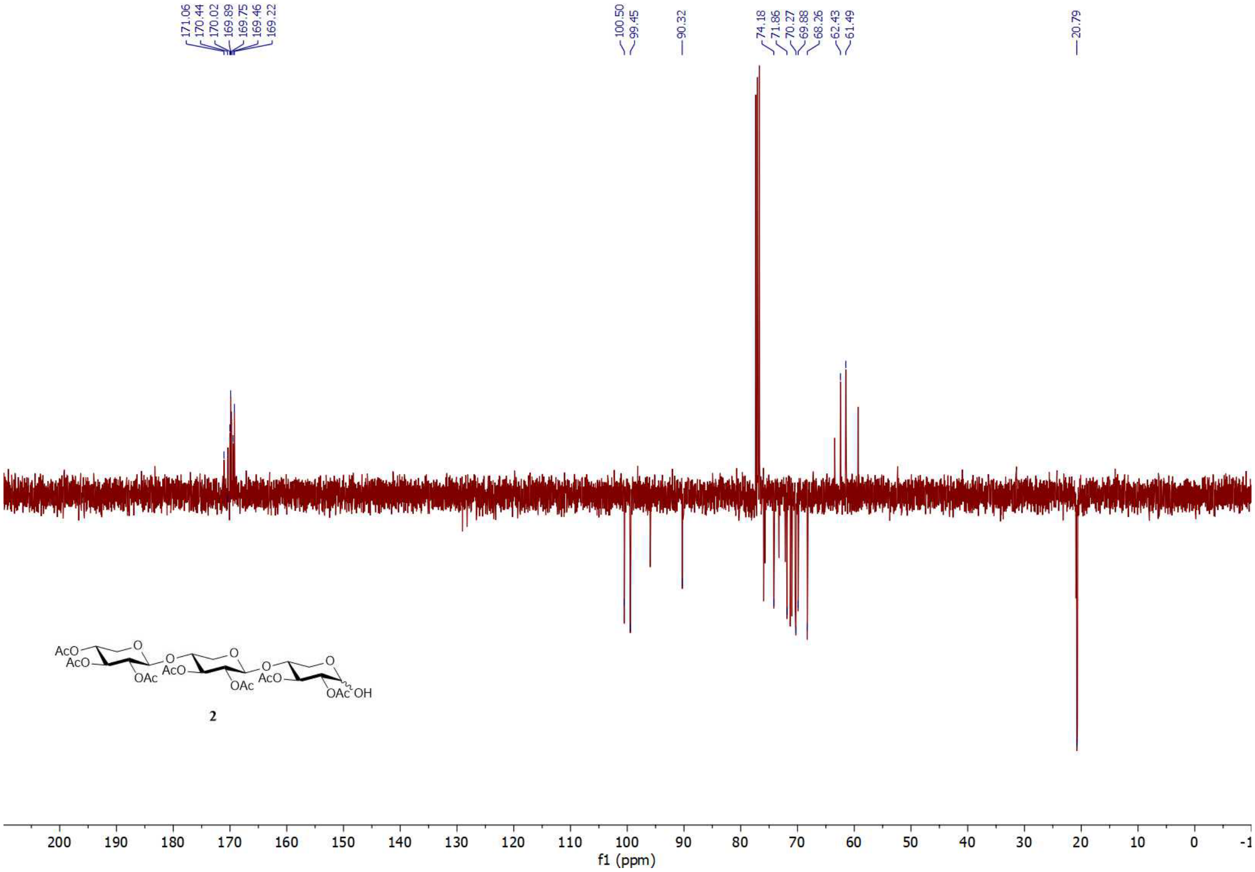

2

* Corresponding author, email: m.ubbink@chem.leidenuniv.nl

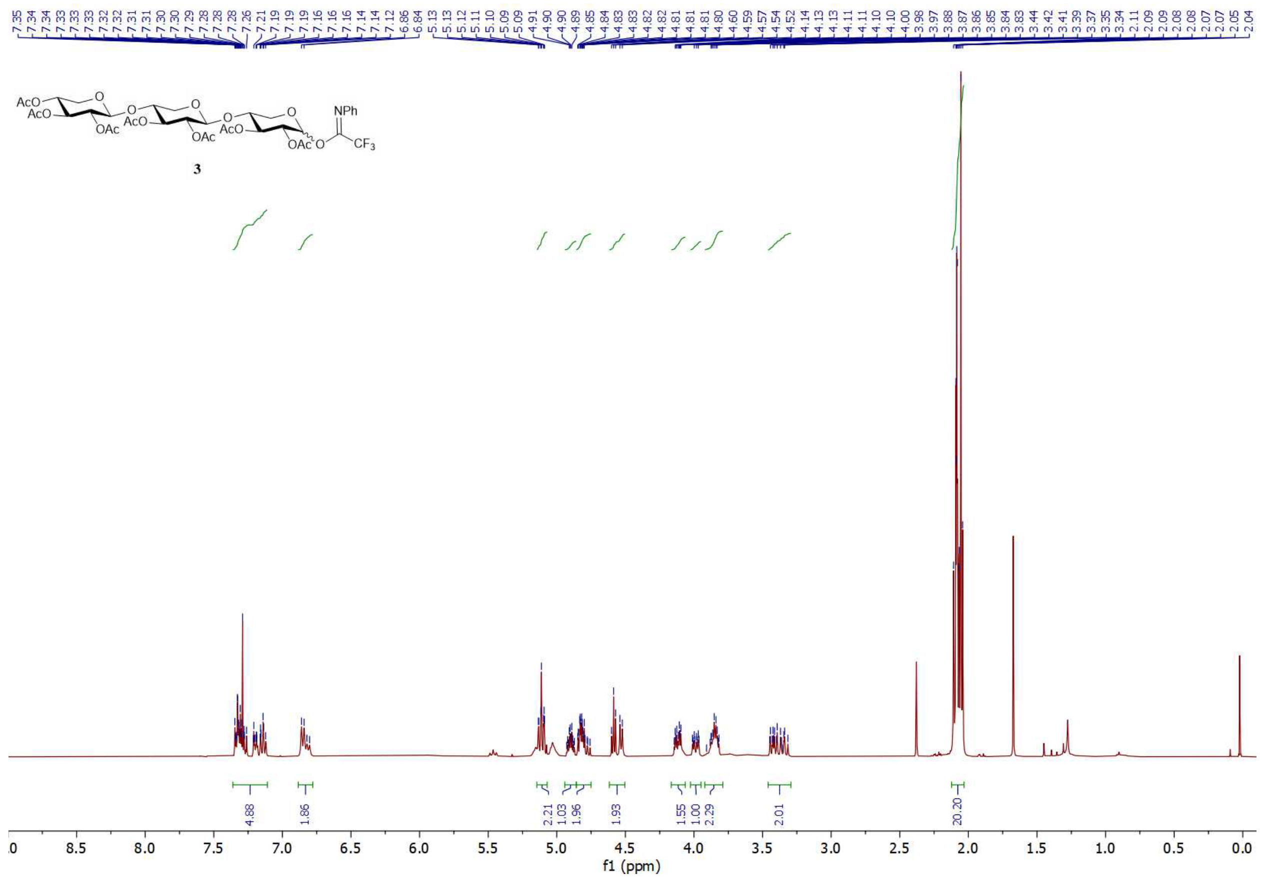

* Corresponding author, email: m.ubbink@chem.leidenuniv.nl

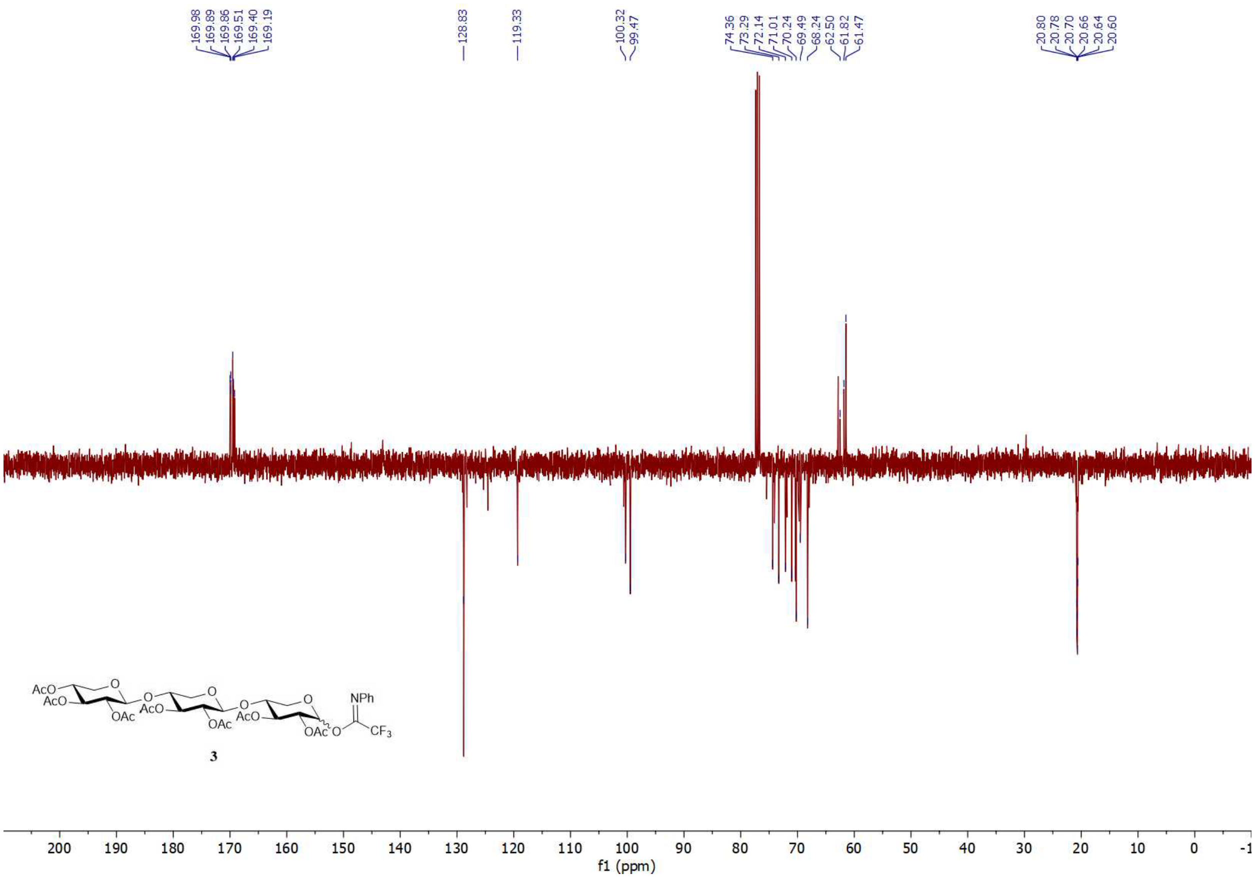

* Corresponding author, email: m.ubbink@chem.leidenuniv.nl

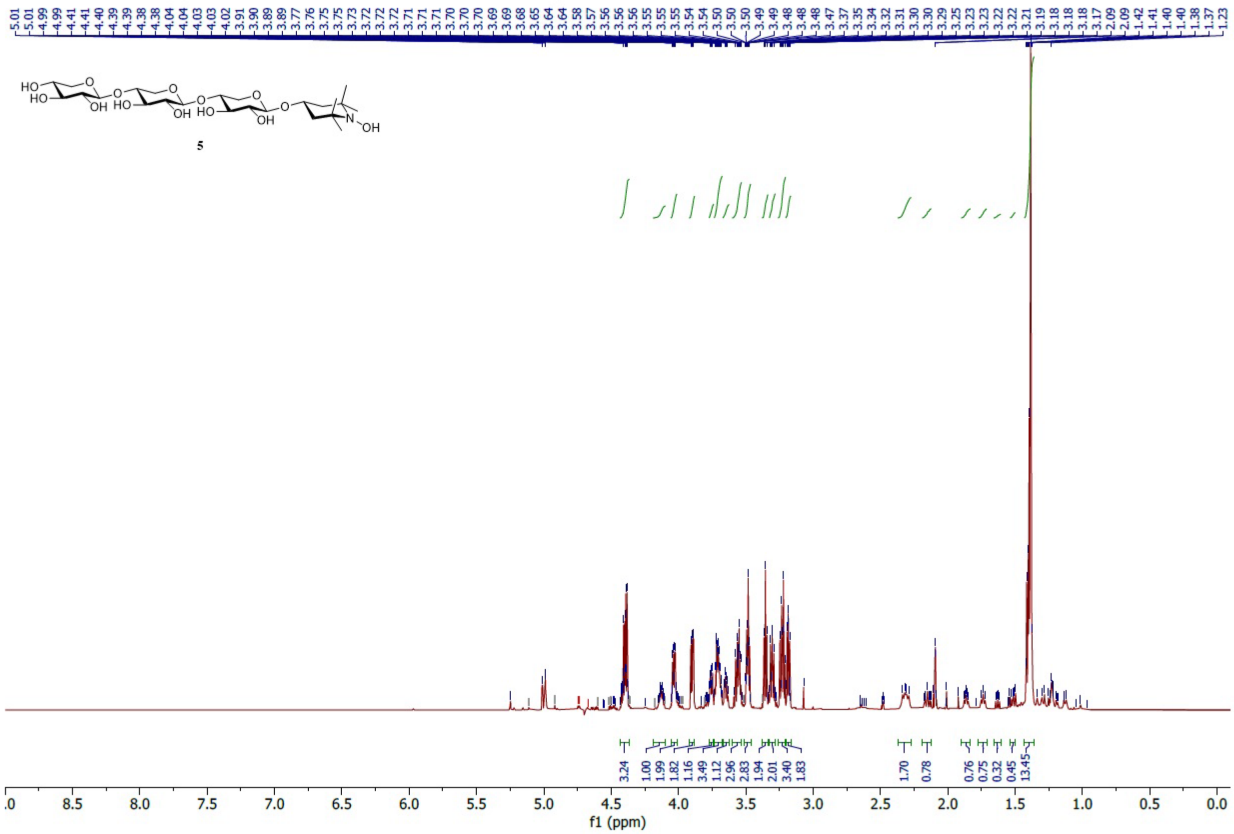

* Corresponding author, email: m.ubbink@chem.leidenuniv.nl

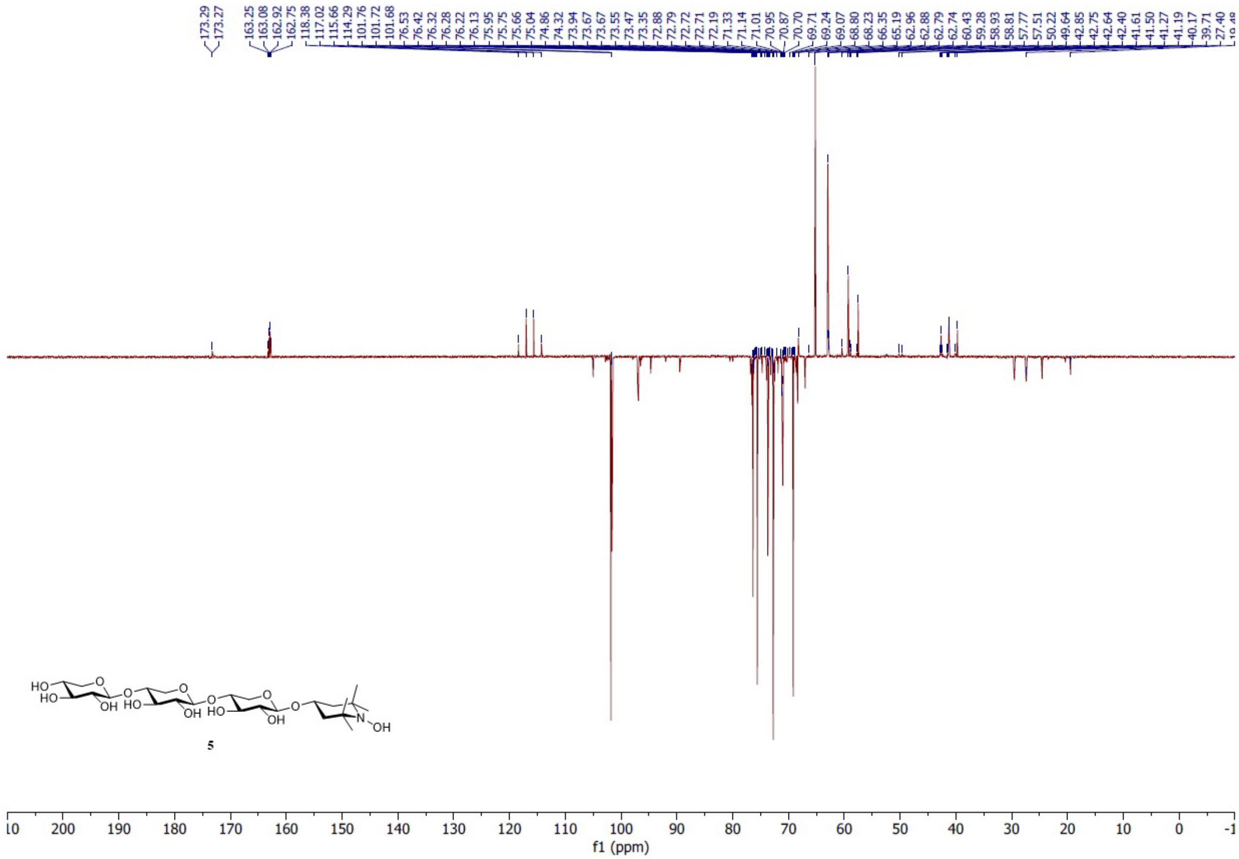

Supplementary references

Smith C D, Bartley J P, Bottle S E, Micallef A S, Reid D A (2000) Electrospray ionization mass spectrometry of stable nitroxide free radicals and two isoindoline nitroxide dimers. J Mass Spectrom 35:607-611

* Corresponding author, email: m.ubbink@chem.leidenuniv.nl
